# Exosomes from human adipose-derived mesenchymal stem cells inhibit production of extracellular matrix in keloid fibroblasts via downregulating transforming growth factor-β2 and Notch-1 expression

**DOI:** 10.1080/21655979.2022.2051838

**Published:** 2022-03-25

**Authors:** Jing Li, Zhiyu Li, Song Wang, Jianhai Bi, Ran Huo

**Affiliations:** a Department of Burn and Plastic Surgery, Shandong Provincial Hospital, Cheeloo College of Medicine, Shandong University, Jinan, Shandong, China; b Department of Burn and Plastic Surgery, Shandong Provincial Hospital Affiliated to Shandong First Medical University, Jinan, Shandong, China

**Keywords:** Adipose-derived mesenchymal stem cells, exosome, keloid fibroblasts, extracellular matrix, TGF-β/smad signaling

## Abstract

Keloids are an excessive tissue response to dermal damage, characterized by uncontrolled growth and a high recurrence rate after various treatments. Abnormalities with the extracellular matrix (ECM) are one of the most important contributing factors to the formation of keloids. Although exosomes from human adipose-derived mesenchymal stem cells (adMSC-Exos) have been shown to promote repair and regeneration in wounds, they have seldom been studied for the treatment of keloids. In this study, we aimed to investigate the effects of adMSC-Exos on ECM remodeling in keloids using both *in vitro* and *ex vivo* models. The results showed that adMSC-Exos inhibited gene and protein expression of collagen I (*COL-1*), collagen III (*COL-3*), fibronectin (*FN*), and α-smooth muscle actin (*α-SMA*) in keloid fibroblasts (KFs). Furthermore, using an *ex vivo* tissue explant model, we found that adMSC-Exos significantly suppressed COL production and disrupted the microvessel stucture. We also demonstrated that adMSC-Exos inhibited the protein expression of Smad3 and Notch-1, and the expression of transforming growth factor β2 (*TGF-β2*) in KFs, and promoted the expression of *TGF-β3*. These findings largely explain the mechanisms underlying the inhibition of ECM production in keloids by adMSC-Exos. In conclusion, our results revealed that adMSC-Exos effectively inhibited the production of ECM in keloids, which provides a new potential alternative for the systemic treatment of keloids.

## Graphical abstract

**Figure:**
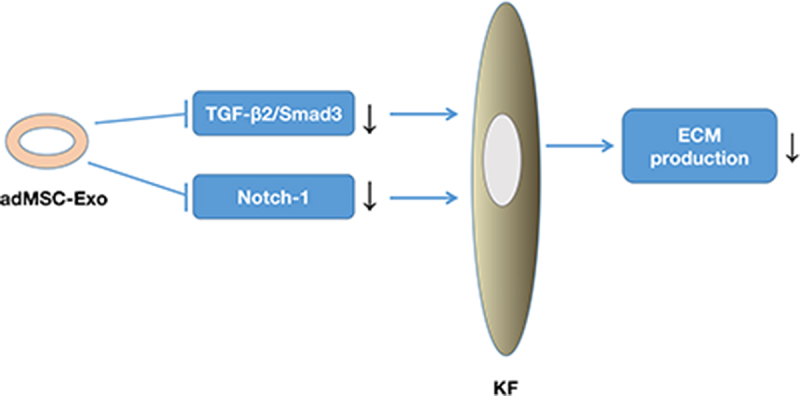

## Introduction

Keloids are benign fibroproliferative skin tumors with unknown pathogenesis that are caused by an abnormal wound healing process following skin injury [1]. Keloids can be accompanied by severe pain, pruritus, and other physical and psychosocial symptoms that affect the quality of life of the patient [2]. To date, none of the available treatments for keloids have been adequately evaluated in high-quality studies, and keloids often recur following any treatment.

An abnormality of the extracellular matrix (ECM) is one of the most important factors in the development of keloids [3]. Compared to normal scars, the collagen bundles in keloids are large, thick, and closely packed randomly to the epidermis, and collagen I (COL-1) and collagen II (COL-2) fibers are haphazardly packed [4]. Studies have shown that collagen synthesis in keloids is approximately 20 times higher than that in normal unscarred skin, and the rate of fibronectin (FN) biosynthesis is four times higher than that of fibroblasts in normal scars [5,6]. Multiple dysregulated signaling pathways have been shown to be involved in ECM overproduction in keloids, such as the transforming growth factor β (TGF-β)/Smad, Wnt/β-catenin, phosphoinositide 3 kinase (PI3K)/AKT/mechanistic target of rapamycin (mTOR), and Notch-1/jagged canonical notch ligand 1 (JAG-1) pathways [1,7,8]. Identifying the exact ECM molecules mediating keloid formation may provide future treatment targets, which could be achieved by promoting the correct balance of collagen ratios or by directly targeting collagen production [3].

Adipose mesenchymal stem cells (adMSCs) are one of the main sources of ECM proteins involved in the maintenance of the skin structure and function. These cells regulate the skin endostasis and healing processes by interacting with skin cells through autocrine or paracrine pathways [9]. adMSCs cultured *in vitro* secrete a variety of bioactive molecules into the culture medium through a paracrine mechanism, resulting in conditioned medium (CM) [10]. Several studies have reported that human adMSC-conditioned medium (adMSC-CM) inhibits the biological activity of keloids [7,11]. Exosomes, as paracrine products of adMSCs originating from the inward budding of the endosomal membrane, are small membraned vesicles (30–150 nm) that carry complex biological information into target cells [12]. Accumulating evidence has shown that human adMSCs exosomes (adMSC-Exos) modulate immune responses and inflammation, promote angiogenesis, accelerate the proliferation and re-epithelialization of skin cells, and regulate collagen remodeling to inhibit scar hyperplasia [13]. Compared to adMSC therapeutics, adMSC-Exos have high stability and are easily stored. However, no studies have demonstrated whether adMSC-Exos regulate collagen remodeling or ECM synthesis and degradation in keloids.

Therefore, in this study, we aimed to explore the effect of adMSC-Exos on ECM remodeling in keloids and antagonizing signaling pathways using *in vitro* and *ex vivo* models, to provide a new idea for the systematic treatment of keloids. Furthermore, we investigated how removing adMSC-Exos from the adMSC-CM affects their inhibitory activity on keloids.

## Materials and methods

### Patients and samples

All specimens used in this study were obtained from patients in the Burn and Plastic Surgery Department of Shandong Provincial Hospital after informed consent was obtained. Human adipose tissue samples were collected from patients undergoing liposuction, and keloid tissue samples grown for over 1 year without infection were collected from patients without a previous history of treatment. All experiments were approved by the Ethics Committee of Shandong University Provincial Hospital.

### adMSCs isolation, culture and identification

Adipose tissue samples were washed several times with phosphate-buffered saline (PBS, Basalmedia, B310KJ) containing penicillin (1000 U/mL, Basalmedia, S110JV) and streptomycin (1000 μg/mL, Basalmedia, S110JV) and then digested with 0.075% type I collagenase (Sigma, C0130) for 40 min. The digested adipose tissue was filtered through a 100-mesh filter and the resulting suspension was centrifuged to obtain cell precipitates. The cells were cultured in Dulbecco’s modified Eagle’s medium (DMEM, Gibco, C11885500BT) containing 10% fetal bovine serum (FBS, Gibco, A31604-02), penicillin (100 U/mL), and streptomycin (100 μg/mL). The cells were subcultured (passaged) when the cellular confluence reached approximately 80% and adMSCs from the third passage (P3) were used in subsequent experiments.

adMSCs identification was performed as previously described by Wang et al. [11]. Briefly, the immunophenotype of P3 adMSCs was determined by evaluating cluster of differentiation 34 (CD34), CD44, CD45, CD105, CD106, and CD166 using flow cytometry, and osteogenic and adipogenic differentiation was performed to confirm their multipotency.

### Keloid fibroblasts (KFs) isolation and culture

Keloid fibroblasts (KFs) were obtained using the explant culture method as previously described [14]. Briefly, the epidermis was removed from keliod tissue samples, which were then minced into 1.5 mm × 1.5 mm × 1.5 mm fragments, equally treated, seeded into 25 cm^2^ culture flasks (Corning, 430,639), and incubated in DMEM containing 20% FBS with 1% penicillin and streptomycin for 1 week. The medium was then replaced with fresh medium containing 10% FBS every 2 days. When more than 80% of the isolated fibroblasts were confluent, they were passaged and KFs from P3 were used for subsequent experiments.

### adMSC-Exos extraction and identification

Exosomes were extracted as previously described [15]. Briefly, the adMSC culture medium was collected, centrifuged at 300 ×* g* for 10 min, and then further centrifuged at 2000 × *g* for 10 min to remove the dead cells. The supernatant was subsequently centrifuged at 10,000 × *g* for 30 min to remove cell debris and the final supernatant was ultracentrifuged at 100,000 × *g* for 70 min to obtain the small vesicles considered as exosomes and the adMSC-Exo-free supernatant (AEFS). The resulting pellets were resuspended in 10 mL PBS, to eliminate contaminating proteins and ultracentrifuged at 100,000 × *g* for 70 min. All procedures were conducted at 4°C. The exosomes were then resuspended in 100 μL PBS to form an exosome suspension. A bicinchoninic acid (BCA) protein assay kit (Solarbio, PC0020-500) was used for exosomes protein concentration quantification, and a microplate reader (Thermo, Multiskan GO, USA) equipped with Multiskan GO 3.1 software (Thermo, USA) for exosomes protein concentration analysis. The exosomes were identified using a transmission electron microscope (TEM, Hitachi HT7800) to confirm their presence and a nanoparticle size detector (Particle Metrix, ZetaView PMX110) to analyze their size.

### Exosome uptake

To confirm that adMSC-Exos could be internalized in KFs, they were labeled using a PKH67 fluorescent cell linker kit (Sigma-Aldrich, MIDI67-1KT) according to the manufacturer’s instructions. The labeled adMSC-Exos were then co-cultured with P3 KFs for 24 h, and images were acquired at 0 and 24 h using a fluorescence microscope (Olympus, IX81) after staining the nucleus with 4’,6-diamidino-2-phenylindole (DAPI, Solarbio, C0065).

### KFs treatment

P3 KFs cultured were grouped into three groups, the first group was treated with 100 μg/mL adMSC-Exos (EXO group), the second group with 100% AEFS (AEFS group), and the third group with a predetermined volume of PBS (PBS group) since the EXO group medium was added with adMSC-Exos resuspended in PBS. All dishes were subjected to separate analyses after 48 h, including quantitative real-time polymerase chain reaction (qPCR), immunofluorescence staining, and western blotting.

### qPCR

Treated KFs were harvested for total RNA extraction using a SteadyPure Universal RNA extraction kit II (Accurate Biology, AG21027) according to the manufacturer’s instructions. Complementary DNA (cDNA) was then synthesized from 500 ng total RNA per sample using an Evo M-MLV RT mix kit (Accurate Biology, AG11728), followed by qPCR using a SYBR Green Pro Taq HS qPCR kit (Accurate Biology, AG11701). The housekeeping gene, glyceraldehyde-3-phosphate dehydrogenase (GAPDH), was used as an internal control and the qPCR primers used are listed in Table 1.

**Table 1.** Primers used in quantitative PCR analysis

Gene	Primer sequence (5’–3’)
*COL-3*	Forward: TGGTGTTGGAGCCGCTGCCA Reverse: CTCAGCACTAGAATCTGTCC
*FN*	Forward: GCCACTGGAGTCTTTACCACA Reverse: CCTCGGTGTTGTAAGGTGGA
*α-SMA*	Forward: CATCATGCGTCTGGATCTGG Reverse: GGACAATCTCACGCTCAGCA
*MMP-1*	Forward: ACAACTGCCACAAGCAATGAG Reverse: CTGTCCCTGAACAGCCCAGTACTTA
*TIMP-1*	Forward: AAGACCTACACTGTTGGCTGTGAG Reverse: GTCCGTCCACAAGCAATGAG
*TGF-β1*	Forward: AAGGACCTCGGCTGGAAGTG Reverse: CCGGGTTATGCTGGTTGTA
*TGF-β2*	Forward: CTGGATGCAGCCTATTGCTT Reverse: TGGTGAGCGGCTCTAAATCT
*TGF-β3*	Forward: AACCTGAGCACCTCCAGGAC Reverse: GCTGCACTTGCAGGATTTG
*GAPDH*	Forward: TCACCATCTTCCAGGAGCG Reverse: CTGCTTCACCACCTTCTTGA

### Immunofluorescence assay

KFs were seeded into six-well plates with sheet glass and pre-cultured in standard culture medium for 24 h. The medium was then replaced, and KFs were continued to be cultured for 48 h according to the above treatments, followed by fixation in 4% paraformaldehyde for 30 min. Primary and secondary antibodies (Table 2) were diluted according to the manufacturer’s instructions. DAPI was used for nuclear counterstaining and images of COL-1-positive (red) or FN-positive (green) and DAPI nuclear-stained (blue) cells were acquired using a fluorescence microscope (Olympus, IX81).

**Table 2.** Antibodies used in Western blotting and immunohistochemical staining

Targets	Source	Species reaction	Usage
COL-1	Proteintech, 14,695-1-AP	Rabbit-anti-human	WB
COL-3	Proteintech, 22,734-1-AP	Rabbit-anti-human	WB
FN	Proteintech, A12932	Rabbit-anti-human	WB
α-SMA	Servicebio, 14,395-1-AP	Rabbit-anti-human	WB
Notch-1	Abcam, ab52627	Rabbit-anti-human	WB
TGF-β1	Abcam, ab215715	Rabbit-anti-human	WB
Smad3	CST, 12,747 T	Rabbit-anti-human	WB
P-Smad3	CST, 12,747 T	Rabbit-anti-human	WB
Smad2	CST, 12,747 T	Rabbit-anti-human	WB
P-Smad2	CST, 12,747 T	Rabbit-anti-human	WB
β-catenin	Abcam, ab32572	Rabbit-anti-human	WB
P-mTOR	Abcam, ab109268	Rabbit-anti-human	WB
GAPDH	CST, 12,747 T	Rabbit-anti-human	WB
β-Actin	Abcam, ab8227	Rabbit-anti-human	WB
Secondary antibody	Abcam, ab288151	Goat-anti-rabbit	WB
COL-1	Proteintech, 14,695-1-AP	Rabbit-anti-human	IHC
FN	Proteintech, A12932	Rabbit-anti-human	IHC
CD31	Servicebio, GB11063-1	Rabbit-anti-human	IHC
CD34	Servicebio, GB11013	Rabbit-anti-human	IHC
Secondary antibody	Servicebio, G1213	Goat-anti-rabbit	IHC
COL-1	Proteintech, 14,695-1-AP	Rabbit-anti-human	IF
FN	Proteintech, A12932	Rabbit-anti-human	IF
Secondary antibody	Servicebio, GB21303 and GB25303	Goat-anti-rabbit	IF

### Western blot analysis

Total protein was extracted using radioimmunoprecipitation assay (RIPA) buffer (Bryotime, P0013B) containing 1% protease inhibitor cocktail (MCE, HY-K0010) and 1% protein phosphatase inhibitor mixture (Solarbio, P1260-1), and quantified using a BCA protein assay kit.Western blot analysis was performed as previously described [16], and detailed information on the primary and secondary antibodies is listed in Table 2.

### *Ex vivo* explant culture of human keloid tissues

Similar to the procedure used in the explant culture method for KF isolation, the epidermis was removed from keloid tissues, which were minced into 3 mm × 3 mm × 2 mm fragments [17]. The fragments were then equally treated and seeded onto three culture dishes (Corning, 430,167) with DMEM containing 10% FBS. After tissue attachment, they were then treated in the same way as the KFs. The medium was replaced with AEFS or DMEM containing 10% FBS, with or without adMSC-Exos. On day 7 after the KFs had migrated from the edge of the tissue, the fragments were collected for the immunohistochemical analysis.

### Immunohistochemical analysis

Keloid explant specimens were fixed in 4% paraformaldehyde at 4°C overnight, embedded in paraffin blocks, and cut into 5 μm thick sections, which were mounted on slides. Then, the sections were incubated with antibodies against CD31 and CD34 (Table 2), followed by 3,3ʹ-diaminobenzidine to visualize the bound antibodies, and the slides were counterstained with hematoxylin. The number of CD31^+^ and CD34^+^ vessels was semiquantified in five randomly selected fields under a microscope.

## Statistical analysis

All data were acquired from more than three independent experiments and are presented as means ± standard deviation (SD). GraphPad Prism 9 software was used to perform all statistical analyses. The Student’s *t*-test was used to analyze the differences among the three groups, and statistical significance was set at P < 0.05.

## Results

Irregularities of theECM play a critical role in the formation of keloids, and we hypothesized that adMSC-Exos, which regulate the repair of wounds would be a potentially effective treatment. Therefore, we evaluated the effects of adMSC-Exos on ECM remodeling in keloids using both *in vitro* and *ex vivo* models.

### Characterization of adMSCs

**Figure 1.**
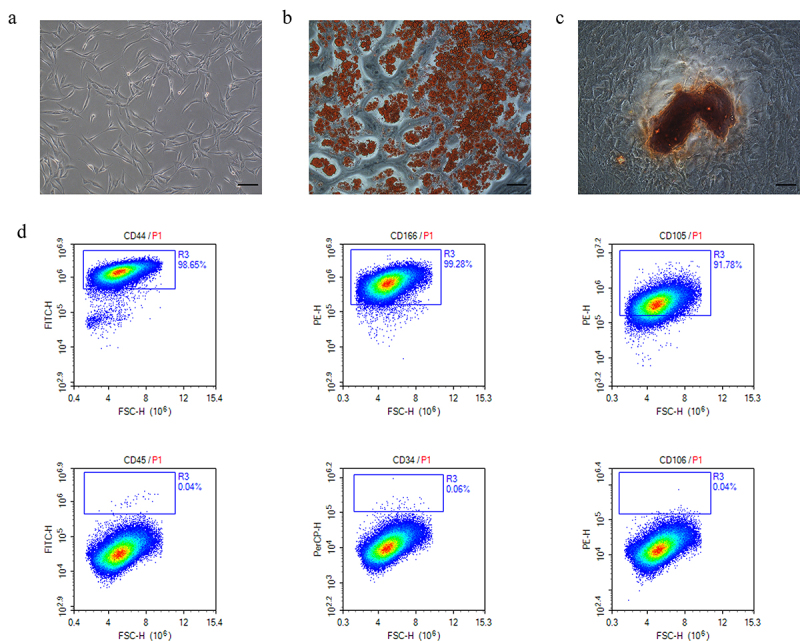
**Characterization of human adipose-derived mesenchymal stem cells (adMSCs). a** adMSCs exhibited fibroblast-like morphology (Scale bar = 100 μm). **b** Cells were induced to differentiate into adipocytes (Scale bar = 100 μm). **c** Brown clumps of calcium salt precipitation on the cell surface after 21 day osteogenic induction culture (Scale bar = 100 μm). **d** Flow cytometric characterization of adMSCs. adMSCs strongly expressed CD44, CD166, and CD105, but not CD 45, CD34, CD106.

Throughout the incubation period, the human adMSCs had a typical fibroblast-like morphology, as previously described [11] (Figure 1a). Adipogenic and osteogenic assays confirmed the multipotent differentiation of the adMSCs. After a 21 day adipogenic induction culture, the oil red O staining displayed the red-stained lipid droplets (Figure 1b). Furthermore, alizarin red S staining revealed brown clumps of calcium salt precipitation on the cell surface after a 21 day osteogenic induction culture (Figure 1c). The flow cytometry results showed that P3 adMSCs highly expressed the surface markers CD105, CD166, and CD44, but not the CD45, CD34, and CD106 markers (Figure 1d). These results suggested that the isolated adMSCs exhibited typical adMSC characteristics.

### Characterization of adMSC-Exos

**Figure 2.**
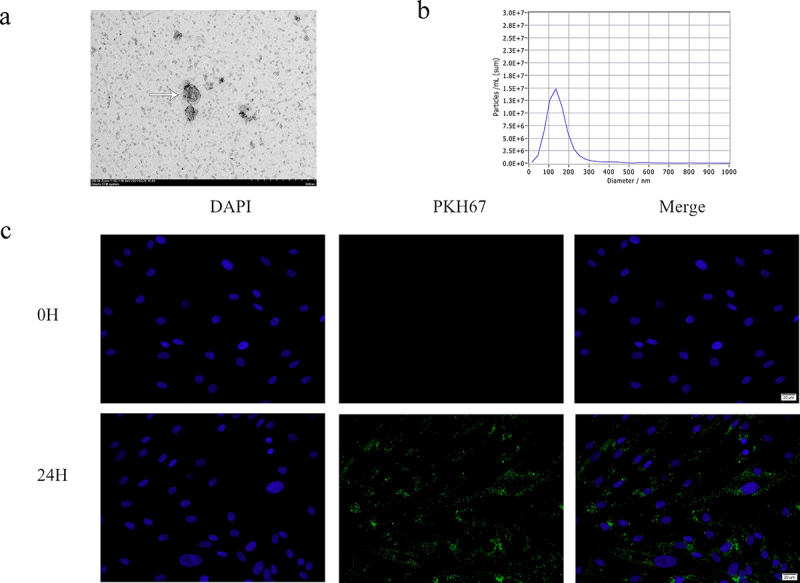
**Characterization of exosomes from adipose-derived mesenchymal stem cells (adMSCs-Exos). a** Electron micrograph of exosomes isolated from adMSC conditional medium (Scale bar = 500 nm). **b** Particle size analysis showed that most of the adMSCs-Exos were 142 nm in diameter. **C** Most adMSC-Exos (green) were taken up by keloid fibroblasts (KFs, blue nucleus) after co-cultured for 24 h (Scale bar = 20 μm).

Typical teato-like exosome particles were visible under the TEM, indicating the successful extraction of exosomes (Figure 2a). The particle size analysis showed that most vesicles were 142 nm in diameter, which was consistent with the size of the exosomes (Figure 2b). To investigate whether adMSC-Exos are taken up by KFs, adMSC-Exos were labeled with PKH67 dye and co-cultured with KFs. The results showed that after 24 h, most adMSC-Exos (green) were distributed around the nucleus (blue), demonstrating that adMSC-Exos were taken up and transferred to the cytoplasm of KFs (Figure 2c). Taken together, these results suggest that adMSC-Exos were successfully isolated and efficiently transferred to KFs.

### adMSC-Exos suppressed expression of ECM-related factors in KFs

**Figure 3.**
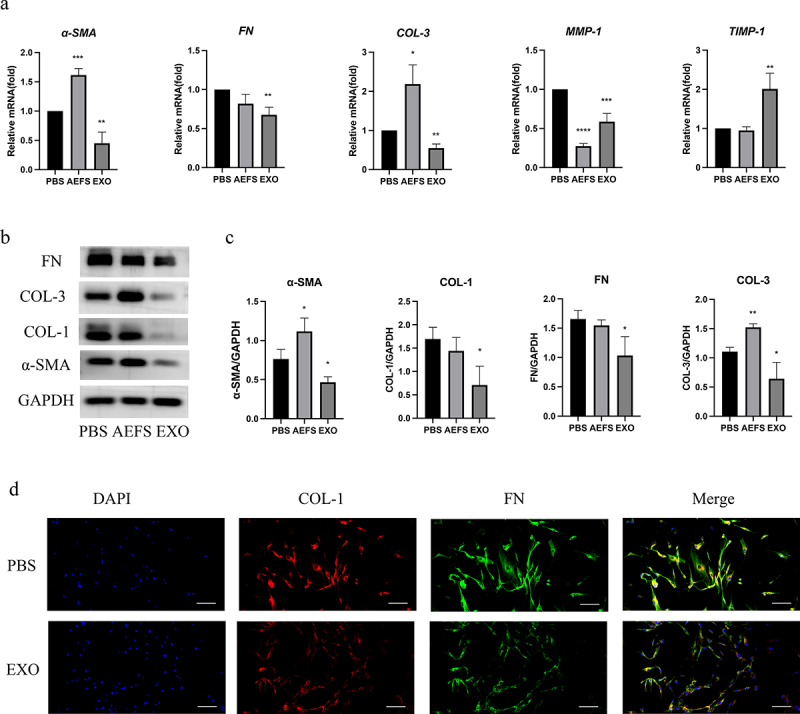
**Antifibrotic effect of adMSC-Exos through inhibition of gene and protein expression of extracellular matrix (ECM)-related factors. a** qPCR analysis of ***F****N, COL-3, α-SMA, MMP-1, and TIMP-1* expression in KFs in the presence or absence of adMSC-Exos after 48 h of treatment. **b** Western blot analysis of COL-1, COL-3, FN, and α-SMA production 48 h post treatment. **c** Semiquantification of western blotting results. **d** Immunofluorescence analysis of COL-1 and FN expression 48 h post treatment (Scale bar = 100 μm). *P < 0.05 vs PBS group, **P < 0.01 vs PBS group, ***P < 0.001 vs PBS group, ****P < 0.0001 vs PBS group.

An *in vitro* cell culture model was used to study the effects of adMSC-Exos on the gene and protein expression of ECM-related factors. Figure 3a shows that treatment of KFs with 100 μg/mL adMSC-Exos for 48 h significantly suppressed the gene expression of *FN, COL-3*, and *α-SMA*, with significant differences between the PBS- and adMSC-Exo-treated groups (P < 0.05). The AEFS group showed a significant increase in the expression of *COL-3* and *α-SMA* (P < 0.05), but no significant effect was found on *FN*.

Consistent with the gene expression analysis results, western blotting also showed that treatment with 100 μg/mL adMSC-Exos for 48 h significantly suppressed the protein production of α-SMA, COL-1, COL-3, and FN (P < 0.05), as displayed in Figure 3b and the semiquantitative analysis revealed significant differences between the PBS- and adMSC-Exo-treated groups (P < 0.05, Figure 3c). The suppressed production of COL-1 and FN proteins was also demonstrated using immunofluorescence staining of KFs treated with 100 μg/mL adMSC-Exos for 48 h (P < 0.05, Figure 3d). KFs cultured in AEFS for 48 h showed increased expression of COL-3 and α-SMA (P < 0.05), whereas no significant effect was observed on the production of FN and COL-1. Figure 3a shows that adMSC-Exos also reduced gene expression of matrix metalloproteinases I (*MMP-1*) and promoted that of tissue inhibitors of matrix metalloproteinases 1 (*TIMP-1*, P < 0.05).

### adMSC-Exos inhibited collagen production and disrupted angiogenesis in keloid tissue explants

**Figure 4.**
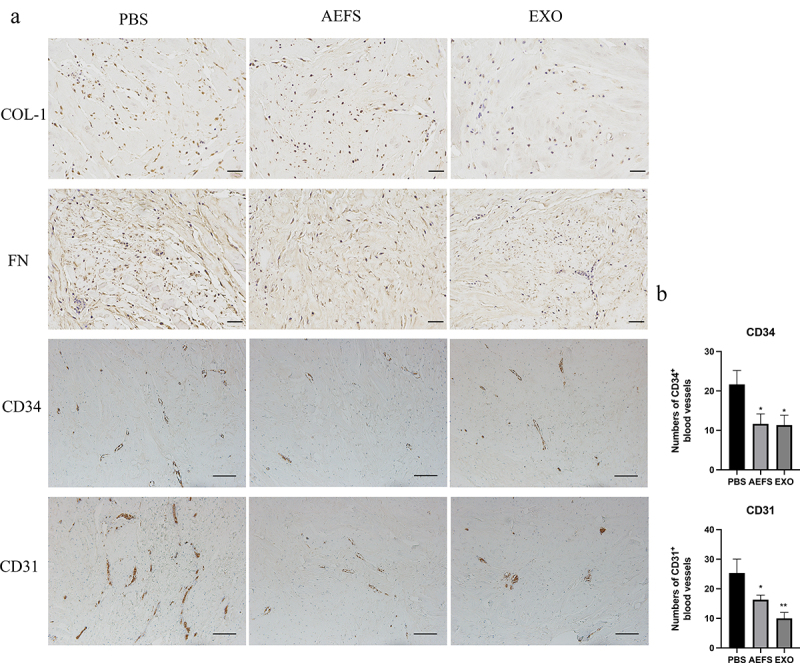
**adMSC-Exos inhibited collagen accumulation and disrupted microvessels in cultured keloid explants. a** Immunohistochemical analysis of COL-1, FN, CD31, and CD34 expression in cultured keloid tissue explants (COL-1 and FN, Scale bar = 100 μm. CD34 and CD31, Scale bar = 200** **μm). b Semiquantitative analysis of CD31^+^ and CD34^+^ microvessel numbers in immunohistochemically stained tissue sections. *P < 0.05 vs PBS group, **P < 0.01 vs PBS group.

Immunohistochemical staining of keloid tissue explants exposed to adMSC-Exos showed a significant decrease in the positive area ratio of COL-1 and FN staining, suggesting that adMSC-Exos significantly decreased their production (Figure 4a). Furthermore, fewer CD34-positive and CD31-positive microvessels were observed in the adMSC-Exo- and AEFS-treated groups than in the PBS group (P < 0.05, Figure 4a, b).

### adMSC-Exos attenuated TGF-β2/Smad3 and Notch-1 signaling in treated KFs

**Figure 5.**
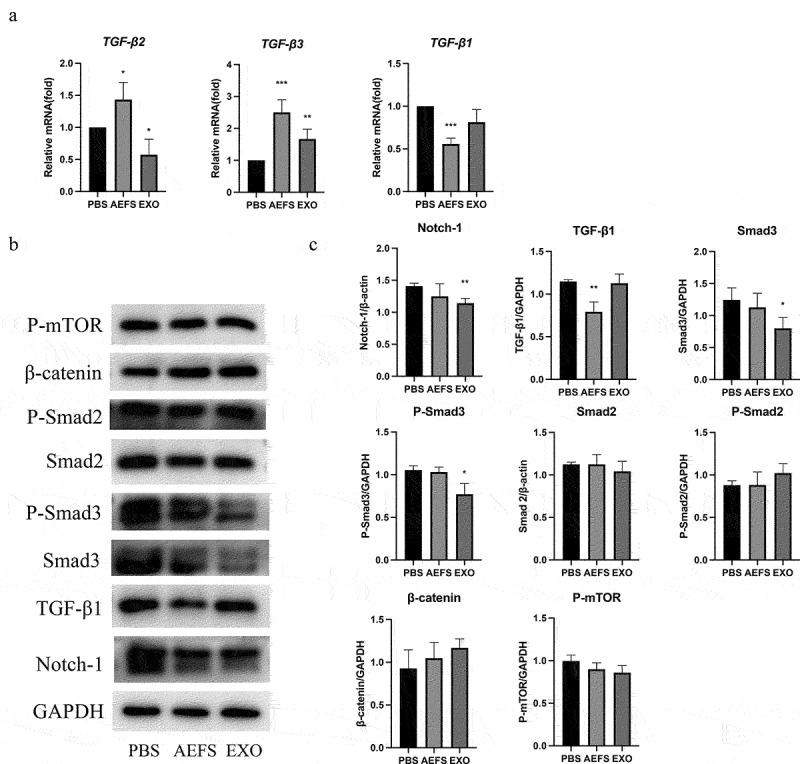
**adMSC-Exos inhibited intracellular signaling pathways *in vitro*. a** qPCR analysis revealed decreased expression of *TGF-β2* and increased that of *TGF-β3* following adMSC-Exos treatment. b Western blotting showed decreased production of Smad3, P-Smad3, and Notch-1 48 h post treatment. c Semiquantitative analysis of the western blotting results. *P < 0.05 vs PBS group, **P < 0.01 vs PBS group, ***P < 0.001 vs PBS group.

To comprehensively elucidate the mechanism underlying adMSC-Exo-induced inhibition of the ECM in keloids, various signaling pathways were investigated using an *in vitro* cell culture model. The qPCR results illustrated in Figure 5a showed that treatment with adMSC-Exos for 48 h decreased the expression of *TGF-β2* and increased that of *TGF-β3* (P < 0.05), but no significant effect was observed on *TGF-β1*. Western blotting results showed that adMSC-Exos significantly inhibited the production of Smad3 protein and its phosphorylation and Notch-1 expression (P < 0.05, Figure 5b, c). KFs cultured in AEFS showed reduced gene and protein expression levels of TGF-β1 (P < 0.05, Figure 5a–c).

## Discussion

Keloids are benign fibroproliferative skin tumors. However, KFs can exhibit a certain degree of some characteristics displayed by cancer cells, particularly the Warburg effect, which increases the survival of KFs under hypoxic conditions [1]. Keloids may cause functional impairment and cosmetic disfigurement and are often associated with low self-reported patient quality of life [18]. Although multiple medical and surgical therapies have been used for the treatment of keloids, none of these treatments has been adequately evaluated in high-quality studies and there is no universally accepted treatment approach. Therefore, new targets and therapeutic approaches are needed.

Recently, stem cell therapy, especially using widely sourced adMSCs, has been the focus of attention because of their pluripotency, self-renewal, and ability to promote the secretion of regenerative cytokines [13]. Exosomes, were one of the components of paracrine and major contributors to stem cell efficacy. In hypertrophic scar, adMSC-Exos have been shown to attenuate collagen deposition, transdifferentiation of fibroblasts to myofibroblasts and proliferative scar formation via the miR-192-5p/IL-17RA/Smad axis [19]. In addition, adMSC-Exos inhibit TGF-β1-induced collagen synthesis in oral mucosal fibroblasts *in vitro* by regulating the p38 MAPK signaling pathway, suggesting that adMSC-Exos may represent a promising strategy for oral submucosal fibrosis treatment by targeting the p38 MAPK signaling pathway [20]. Keloids are fibrotic diseases in which abnormal ECM reconstruction during wound healing contributes to their formation [3]. Existing views suggest that promoting the correct balance of ECM ratios or targeting collagen production may be a future treatment target of keloids. Taken together, in the present study, we detected the effect of adMSC-Exos on ECM remodeling in keloids and further explored the underlying mechanisms. The results demonstrated that adMSC-Exos significantly inhibited the gene expression of *COL-3, FN*, and *α-SMA*, and their protein production *in vitro* (Figure 3). Reduced collagen accumulation was confirmed using an *ex vivo* model (Figure 4). This suggests that adMSC-Exos have the antifibrotic effect through inhibition of gene and protein expression of ECM-related factors, which are expected to be a new potential alternative for the systemic treatment of keloids.

MMP-1, one of the most important collagenases [3], has been shown to be involved in the progression of multiple cancers. In colorectal cancer, MMP1 derived from tumor-associated macrophages activates MAPK/Erk signaling pathway through paracrine PAR1 activation, ultimately facilitating colon cancer cells proliferation [21]. In head and neck squamous cell carcinoma (HNSCC), PLAU1 facilitates HNSCC cell proliferation, invasion, and metastasis via interaction with MMP-1 [22]. In this study, we found that adMSC-Exos significantly suppressed the gene expression of *MMP-1*. This effect may have been due to the adMSC-Exo-mediated enhancement of the expression of *TIMP-1* (Figure 3a), which specifically targets MMP-1 [3]. A decrease in MMP-1 partially reduces matrix degradation, which is necessary for cell migration [17].

The center of the keloid tissue mass often exhibits hypoxia due to capillary obstruction caused by excessive collagen and endothelial cells deposition [23]. The margins of keloids contain active fibroblasts that invade the surrounding tissue and are well vascularized through angiogenesis to maintain the oxygen and nutrient supply required to fuel their invasiveness [1]. In the present study, the inhibitory effect of adMSC-Exos was also supported by decreased CD31-positive and CD34-positive microvessels in an *ex vivo* model, suggesting that adMSC-Exos inhibited the invasiveness of KFs.

TGF-β is a cytokine that has been widely studied in keloid pathogenesis because of its pivotal role in modulating keloid fibrosis [24]. The TGF-β family, including the principal mammalian forms TGF-β1, TGF-β2, and TGF-β3, contributes to the normal wound healing process and is associated with a variety of fibrotic diseases [6]. TGF-β1 and TGF-β2 are among the most important stimulators of ECM synthesis, not only by stimulating collagen synthesis but also by preventing its degradation. In contrast, TGF-β3 has been found to reduce connective tissue deposition [3]. TGF-β1 and TGF-β2 are overexpressed in keloid-derived fibroblasts, whereas TGF-β3 mRNA expression is significantly reduced [25]. Therefore, the imbalance between TGF-β1, TGF-β2, and TGF-β3 is largely responsible for abnormal ECM in keloids. In this study, adMSC-Exos were found to downregulate the gene expression of *TGF-β2* and up-regulate the expression of gene *TGF-β3*, but had no significant effect on gene *TGF-β1*. Furthermore, the results shown in Figure 5 demonstrate that adMSC-Exos inhibited Smad3 and Notch-1 expression, suggesting the involvement of TGF-β2/Smad3 pathway and Notch-1 signaling inhibition in the inhibitory effect of adMSC-Exos on KFs.

In addition, our results indicated that the AEFS retained biological activity, including regulation of TGF-β family and reduction in CD31-positive and CD34-positive microvessels in an *ex vivo* model. These findings suggested the potential usefulness of AEFS in relevant research models.

## Conclusion

The findings of this study demonstrated that adMSC-Exos inhibited ECM deposition in keloids, which may have been mediated by inhibition of the TGF-β2/Smad3 and Notch-1 signaling pathways. Although the detailed underlying mechanisms still need to be further explored, the results of the current study support the conclusion that adMSC-Exo is a likely promising candidate for the treatment of keloids.

## Supplementary Material

Supplemental Material
