# Supplementary material for: Exosomes from human adipose-derived mesenchymal stem cells inhibit production of extracellular matrix in keloid fibroblasts via downregulating transforming growth factor-β2 and Notch-1 expression
Source: Bioengineered. 2022 Mar 25;13(4):8515–25. doi: 10.1080/21655979.2022.2051838 (PMC9161879; doi:10.1080/21655979.2022.2051838)
Supplement: Supplemental Material [file KBIE_A_2051838_SM7045.zip › supplementary document/ethical approvement/2020-050.pdf]

山东省立医院涉及人的生物医学研究伦理委员会
快速伦理审查批件

省医伦批第（SWYX:NO. 2022-050）

<table>
<tr><td>项目名称</td><td colspan="3">人脂肪间充质干细胞来源的外泌体对瘢痕疙瘩细胞外基质重塑的调控作用及其机制研究</td></tr>
<tr><td>项目负责人</td><td>霍然</td><td>承担科室</td><td>烧伤整形外科</td></tr>
<tr><td>项目类型</td><td colspan="3">研究者发起的项目</td></tr>
<tr><td>审查文件</td><td colspan="3">1.伦理审查申请；
2.研究方案；
3.豁免知情同意申请；
4.主要研究者简历。</td></tr>
<tr><td>审查时间</td><td>2022 年 02 月 15 日</td><td>审查方式</td><td>快速审查</td></tr>
<tr><td colspan="2">伦理审查意见</td><td colspan="2">同意</td></tr>
<tr><td colspan="4">审批意见
1.经本伦理委员会审查，同意按审查文件开展研究。意见和建议：■无　　□有
2.伦理委员会对该研究实施过程的年度/定期跟踪审查：■是　　□否
审查期限为研究批准之日起：□三个月　□六个月　■十二个月
3.伦理委员会有权根据实际进展情况改变年度/定期跟踪审查期限。
4.自批准之日起半年内项目未启动，该批件自动失效。

山东省立医院涉及人的生物医学研究伦理委员会
（盖章）
2022 年 02 月 15 日</td></tr>
</table>
