# Supplementary figures and images for: Exosomes from human adipose-derived mesenchymal stem cells inhibit production of extracellular matrix in keloid fibroblasts via downregulating transforming growth factor-β2 and Notch-1 expression
Source: Bioengineered. 2022 Mar 25;13(4):8515–25. doi: 10.1080/21655979.2022.2051838 (PMC9161879; doi:10.1080/21655979.2022.2051838)

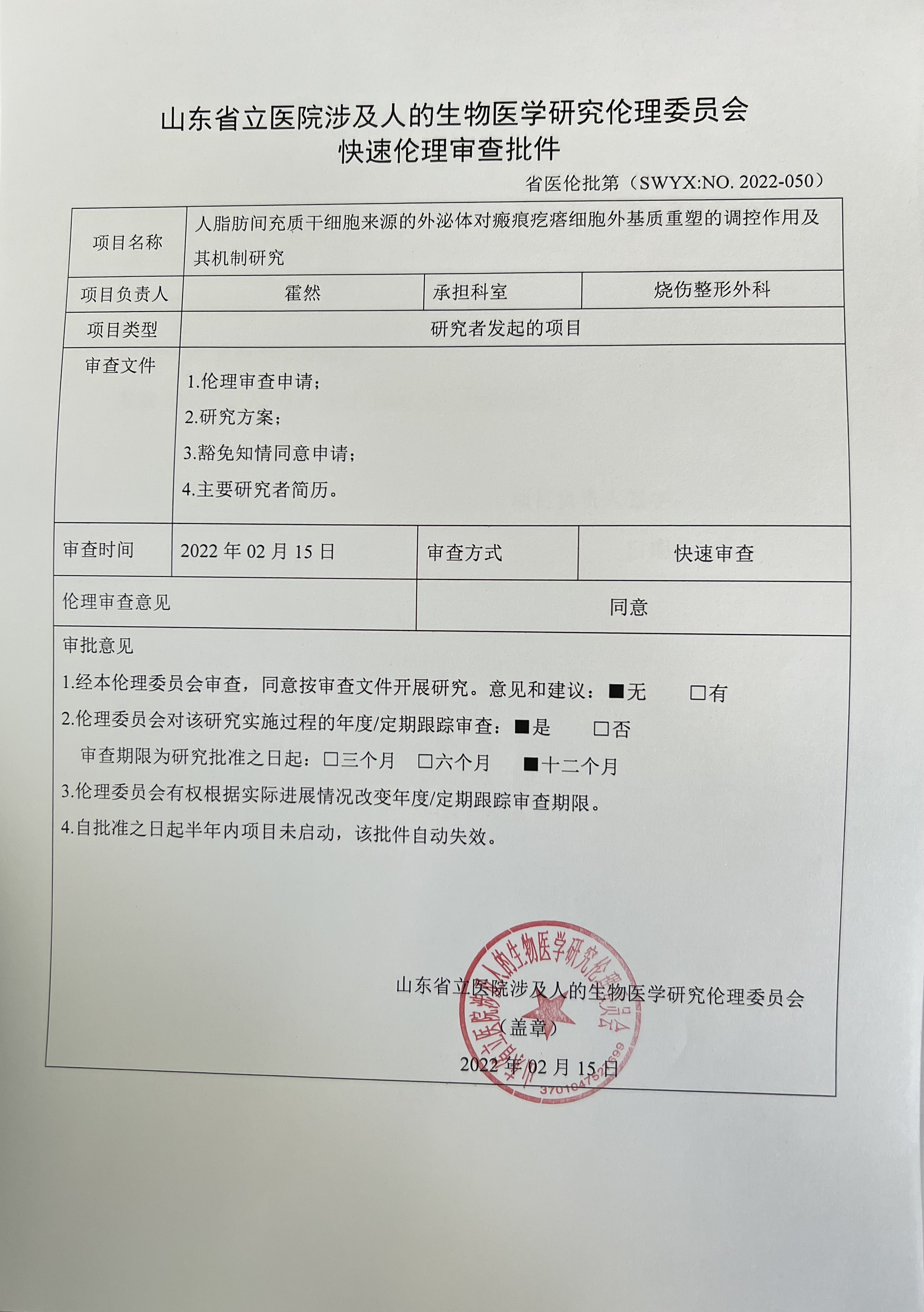

Supplement: Supplemental Material [file KBIE_A_2051838_SM7045.zip › supplementary document/ethical approvement/2022-050.jpeg]

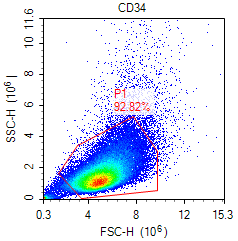

Supplement: Supplemental Material [file KBIE_A_2051838_SM7045.zip › supplementary document/flow cytometry raw data/CD34/σ¢╛1.png]

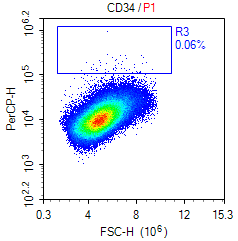

Supplement: Supplemental Material [file KBIE_A_2051838_SM7045.zip › supplementary document/flow cytometry raw data/CD34/σ¢╛2.png]

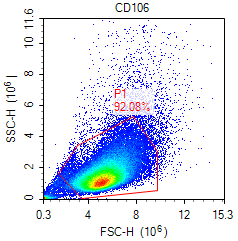

Supplement: Supplemental Material [file KBIE_A_2051838_SM7045.zip › supplementary document/flow cytometry raw data/CD106/σ¢╛1.png]

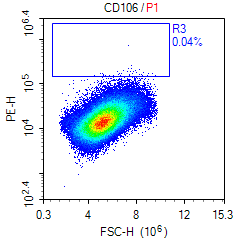

Supplement: Supplemental Material [file KBIE_A_2051838_SM7045.zip › supplementary document/flow cytometry raw data/CD106/σ¢╛2.png]

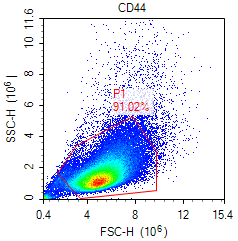

Supplement: Supplemental Material [file KBIE_A_2051838_SM7045.zip › supplementary document/flow cytometry raw data/CD44/σ¢╛1.png]

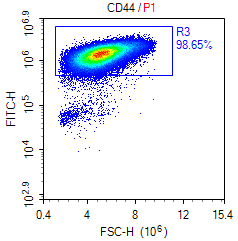

Supplement: Supplemental Material [file KBIE_A_2051838_SM7045.zip › supplementary document/flow cytometry raw data/CD44/σ¢╛2.png]

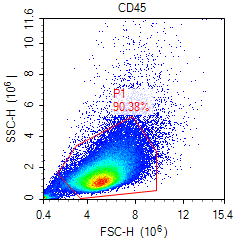

Supplement: Supplemental Material [file KBIE_A_2051838_SM7045.zip › supplementary document/flow cytometry raw data/CD45/σ¢╛1.png]

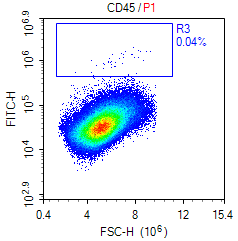

Supplement: Supplemental Material [file KBIE_A_2051838_SM7045.zip › supplementary document/flow cytometry raw data/CD45/σ¢╛2.png]

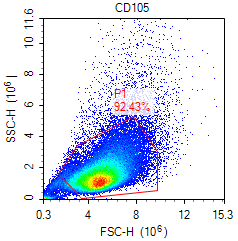

Supplement: Supplemental Material [file KBIE_A_2051838_SM7045.zip › supplementary document/flow cytometry raw data/CD105/σ¢╛1.png]

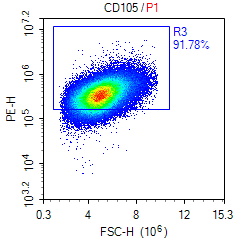

Supplement: Supplemental Material [file KBIE_A_2051838_SM7045.zip › supplementary document/flow cytometry raw data/CD105/σ¢╛2.png]

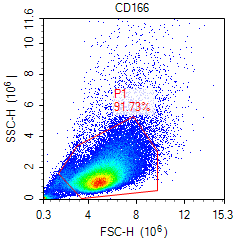

Supplement: Supplemental Material [file KBIE_A_2051838_SM7045.zip › supplementary document/flow cytometry raw data/CD166/σ¢╛1.png]

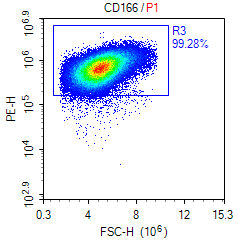

Supplement: Supplemental Material [file KBIE_A_2051838_SM7045.zip › supplementary document/flow cytometry raw data/CD166/σ¢╛2.png]

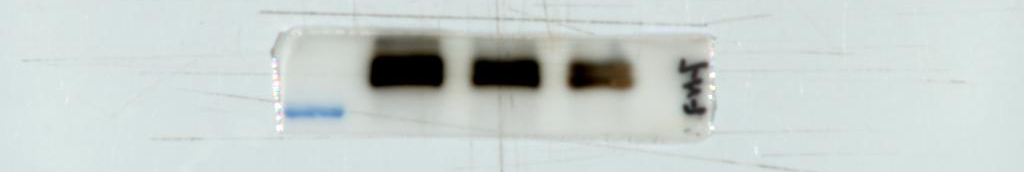

Supplement: Supplemental Material [file KBIE_A_2051838_SM7045.zip › supplementary document/western blot images /Figure 3B/FN.jpg]

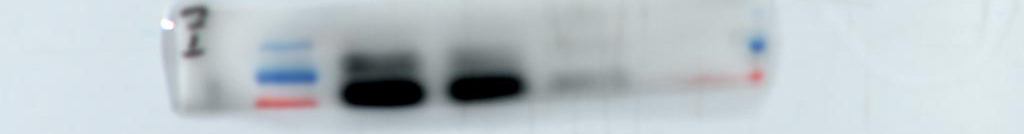

Supplement: Supplemental Material [file KBIE_A_2051838_SM7045.zip › supplementary document/western blot images /Figure 3B/COL-1.jpg]

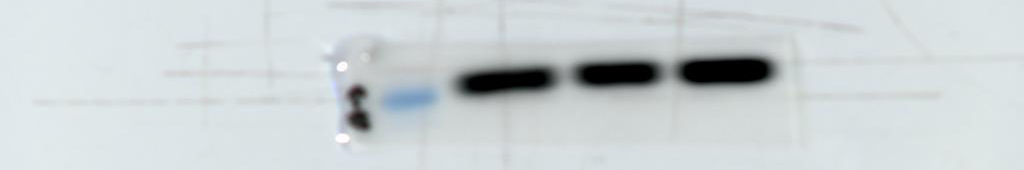

Supplement: Supplemental Material [file KBIE_A_2051838_SM7045.zip › supplementary document/western blot images /Figure 3B/GAPDH.jpg]

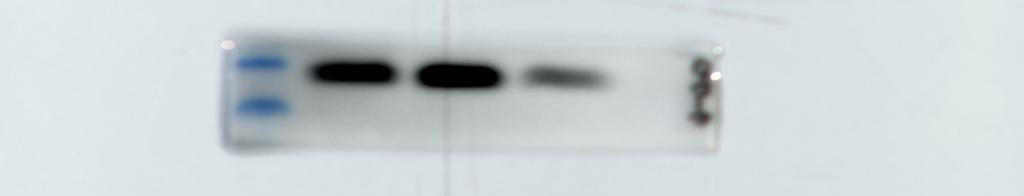

Supplement: Supplemental Material [file KBIE_A_2051838_SM7045.zip › supplementary document/western blot images /Figure 3B/ ╬▒-SMA.jpg]

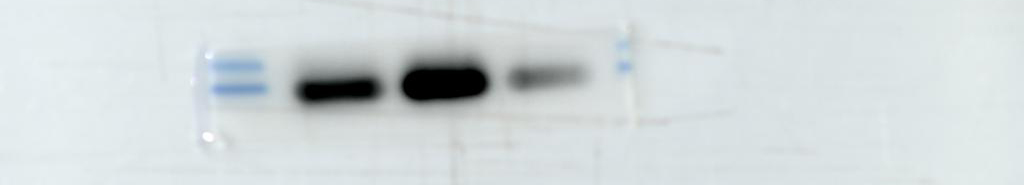

Supplement: Supplemental Material [file KBIE_A_2051838_SM7045.zip › supplementary document/western blot images /Figure 3B/COL-3.jpg]

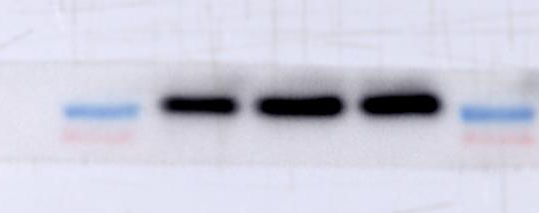

Supplement: Supplemental Material [file KBIE_A_2051838_SM7045.zip › supplementary document/western blot images /Figure 5B/╬▓-catenin.jpg]

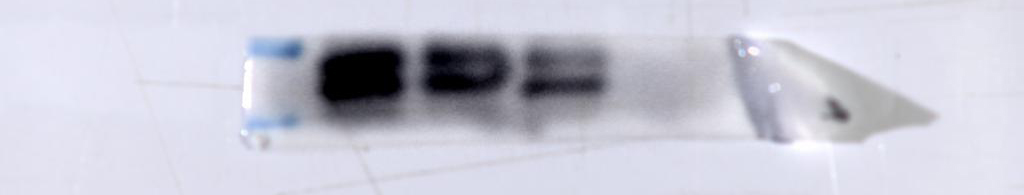

Supplement: Supplemental Material [file KBIE_A_2051838_SM7045.zip › supplementary document/western blot images /Figure 5B/P-Smad3.jpg]

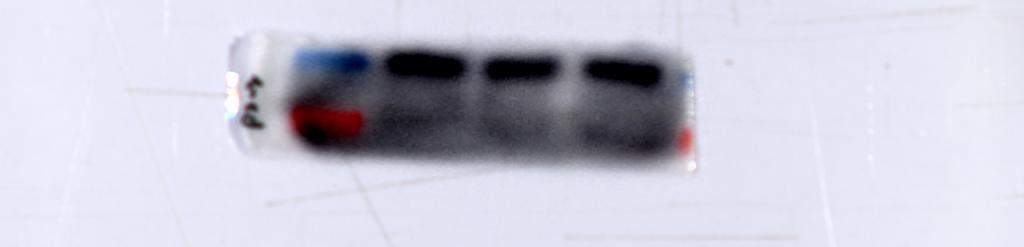

Supplement: Supplemental Material [file KBIE_A_2051838_SM7045.zip › supplementary document/western blot images /Figure 5B/P-Smad2.jpg]

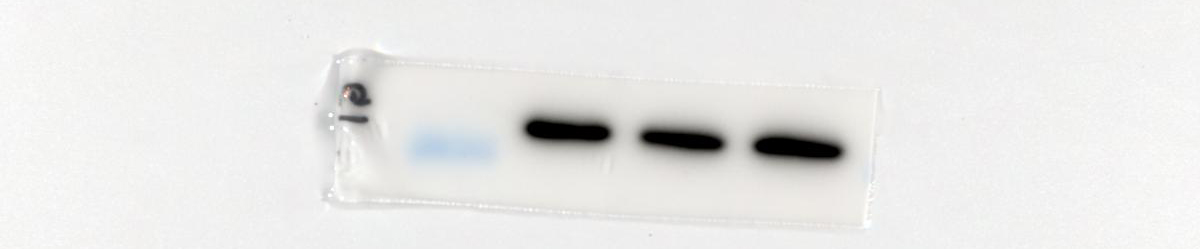

Supplement: Supplemental Material [file KBIE_A_2051838_SM7045.zip › supplementary document/western blot images /Figure 5B/GAPDH.jpg]

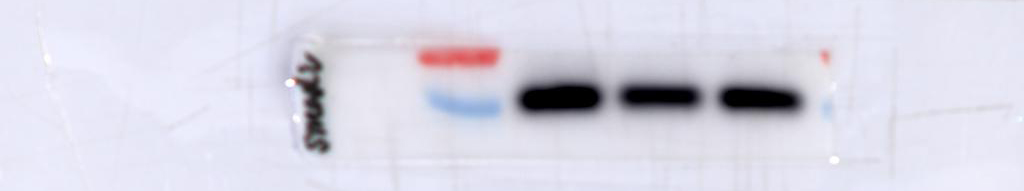

Supplement: Supplemental Material [file KBIE_A_2051838_SM7045.zip › supplementary document/western blot images /Figure 5B/Smad2.jpg]

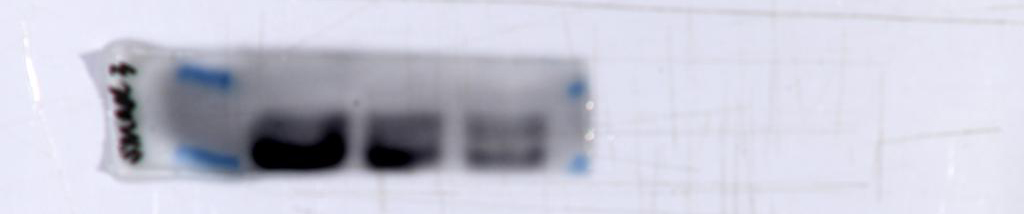

Supplement: Supplemental Material [file KBIE_A_2051838_SM7045.zip › supplementary document/western blot images /Figure 5B/Smad3.jpg]

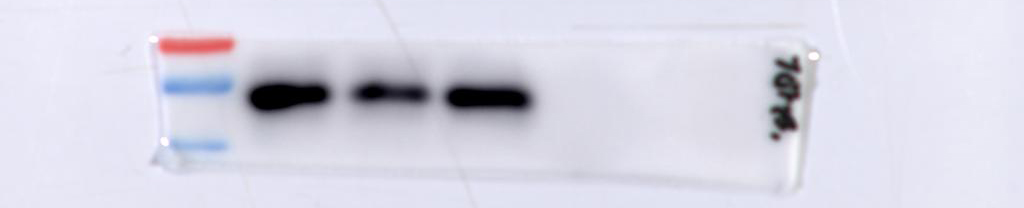

Supplement: Supplemental Material [file KBIE_A_2051838_SM7045.zip › supplementary document/western blot images /Figure 5B/TGF ╬▓1.jpg]

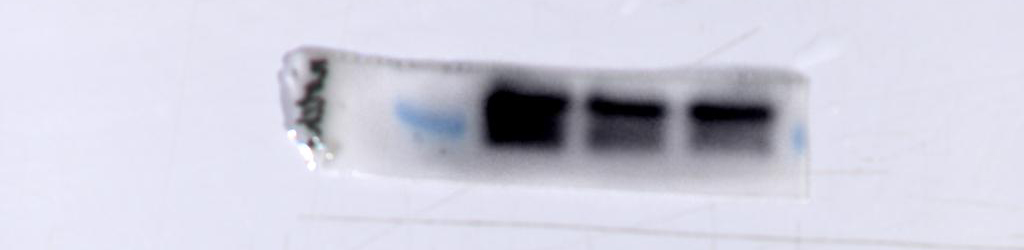

Supplement: Supplemental Material [file KBIE_A_2051838_SM7045.zip › supplementary document/western blot images /Figure 5B/Notch-1.jpg]

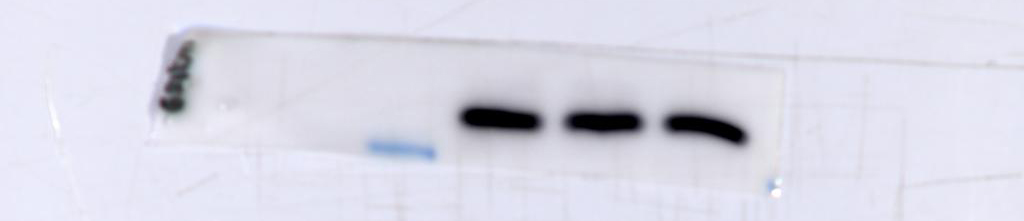

Supplement: Supplemental Material [file KBIE_A_2051838_SM7045.zip › supplementary document/western blot images /Figure 5B/P-mTOR.jpg]

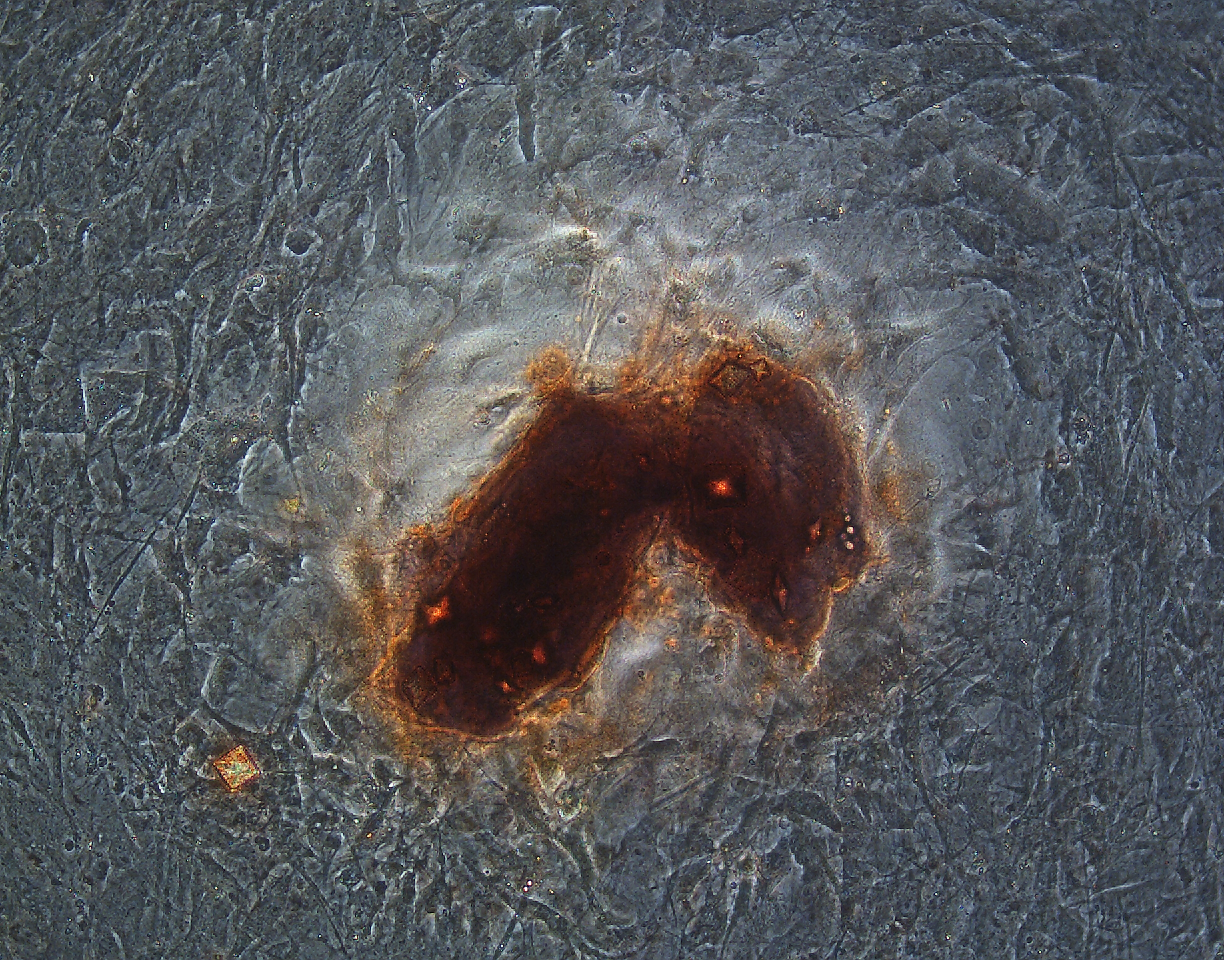

Supplement: Supplemental Material [file KBIE_A_2051838_SM7045.zip › supplementary document/images /Figure 1/Figure 1C.tif]

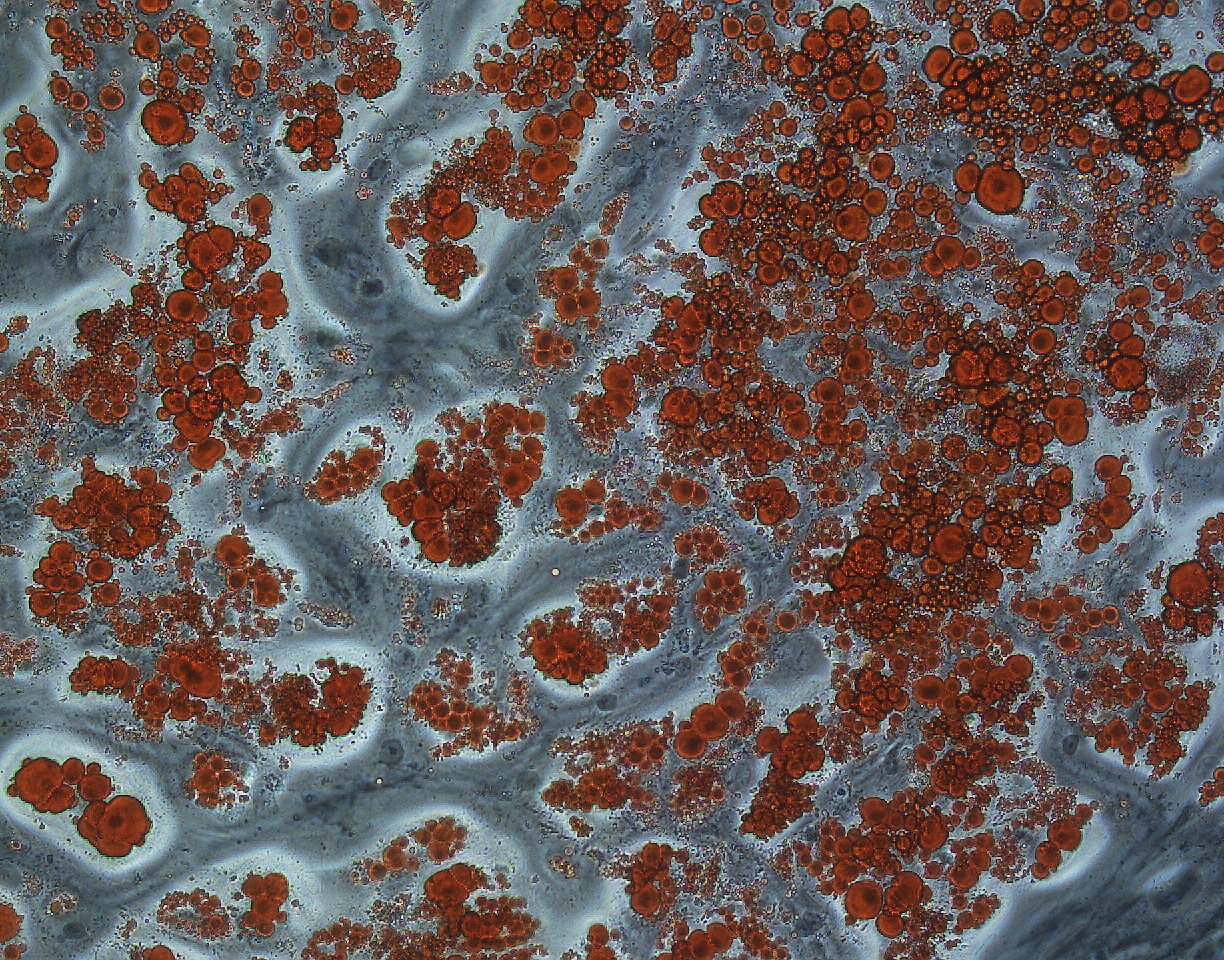

Supplement: Supplemental Material [file KBIE_A_2051838_SM7045.zip › supplementary document/images /Figure 1/Figure 1B.tif]

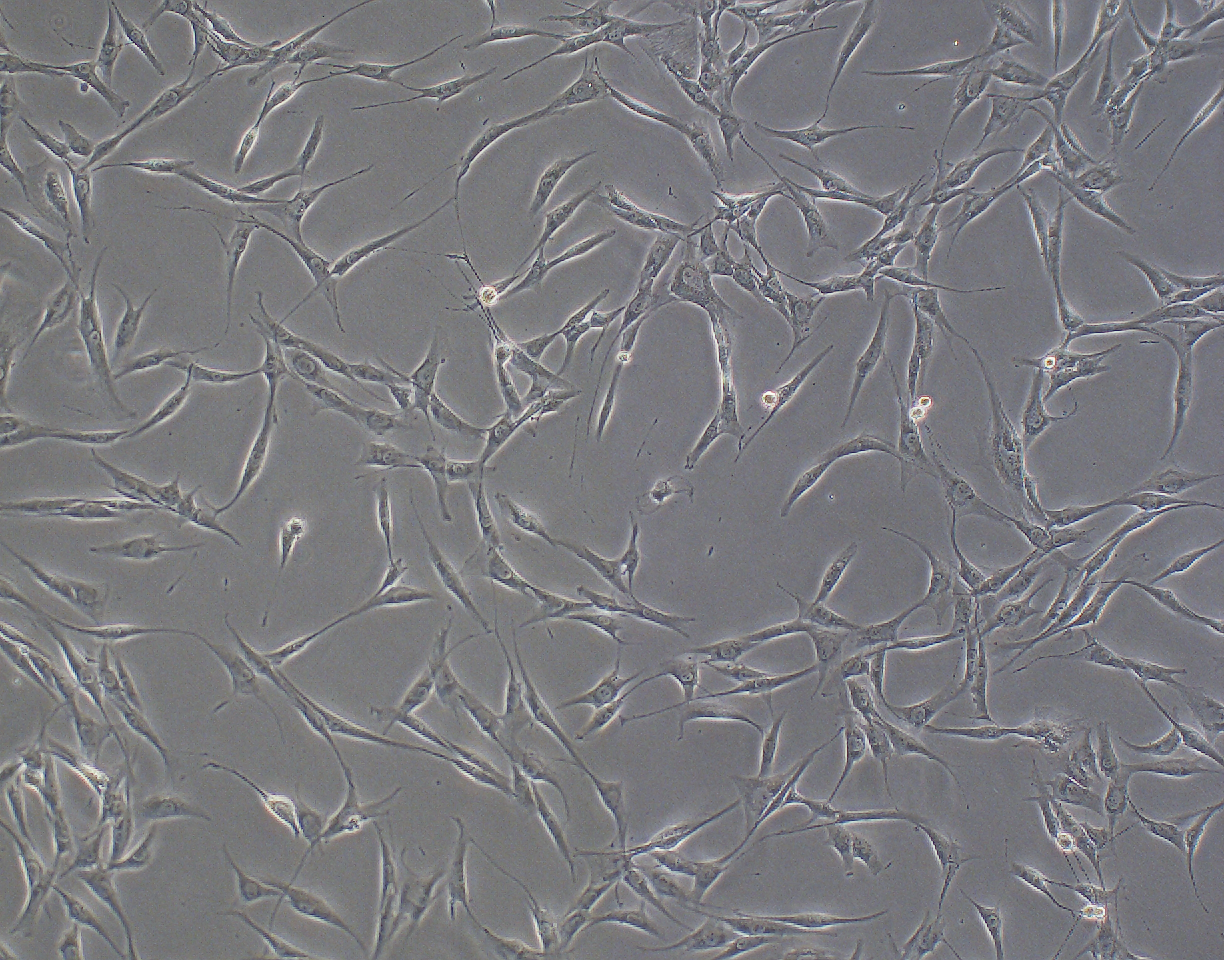

Supplement: Supplemental Material [file KBIE_A_2051838_SM7045.zip › supplementary document/images /Figure 1/figure 1A .tif]

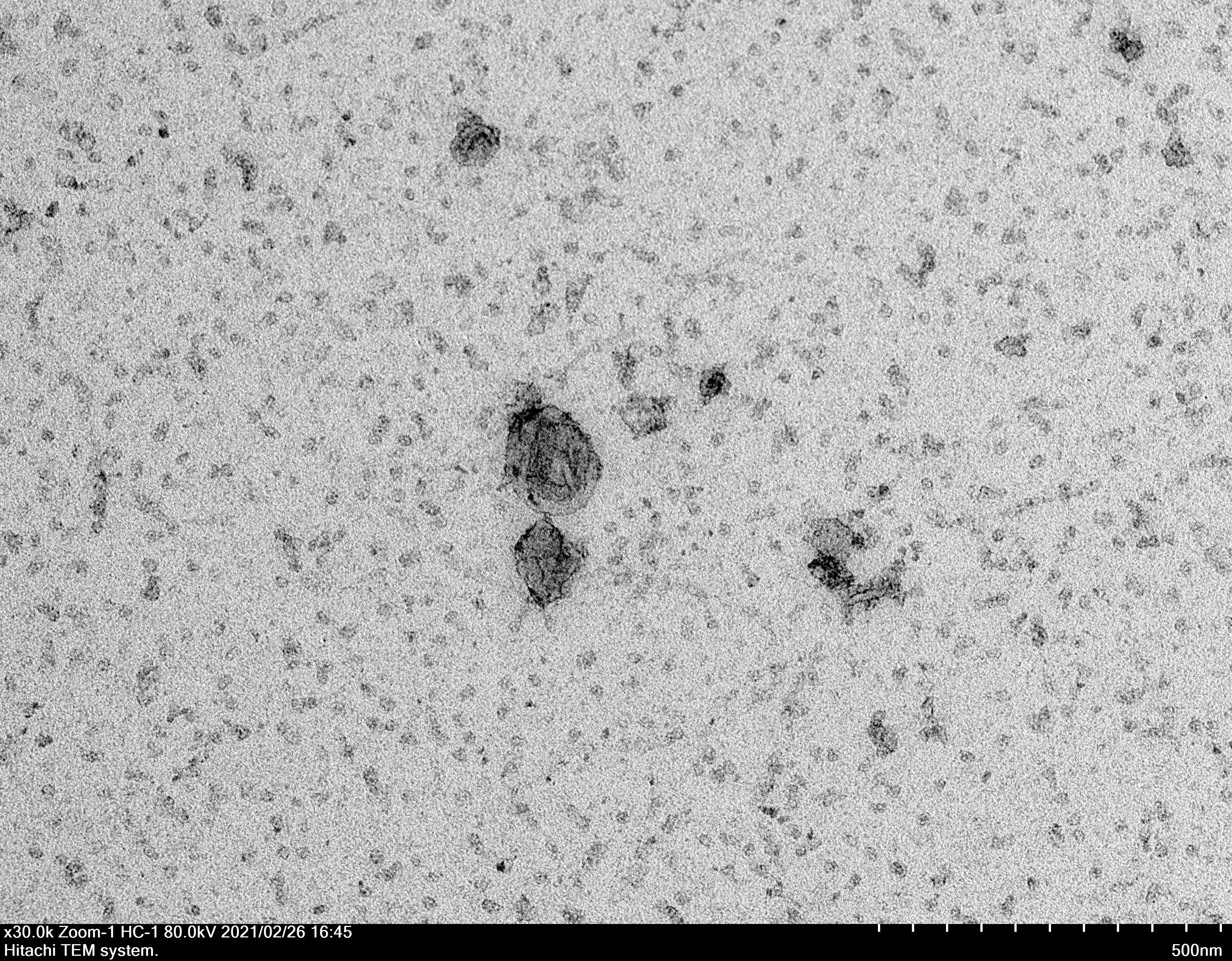

Supplement: Supplemental Material [file KBIE_A_2051838_SM7045.zip › supplementary document/images /Figure 2/A.tif]

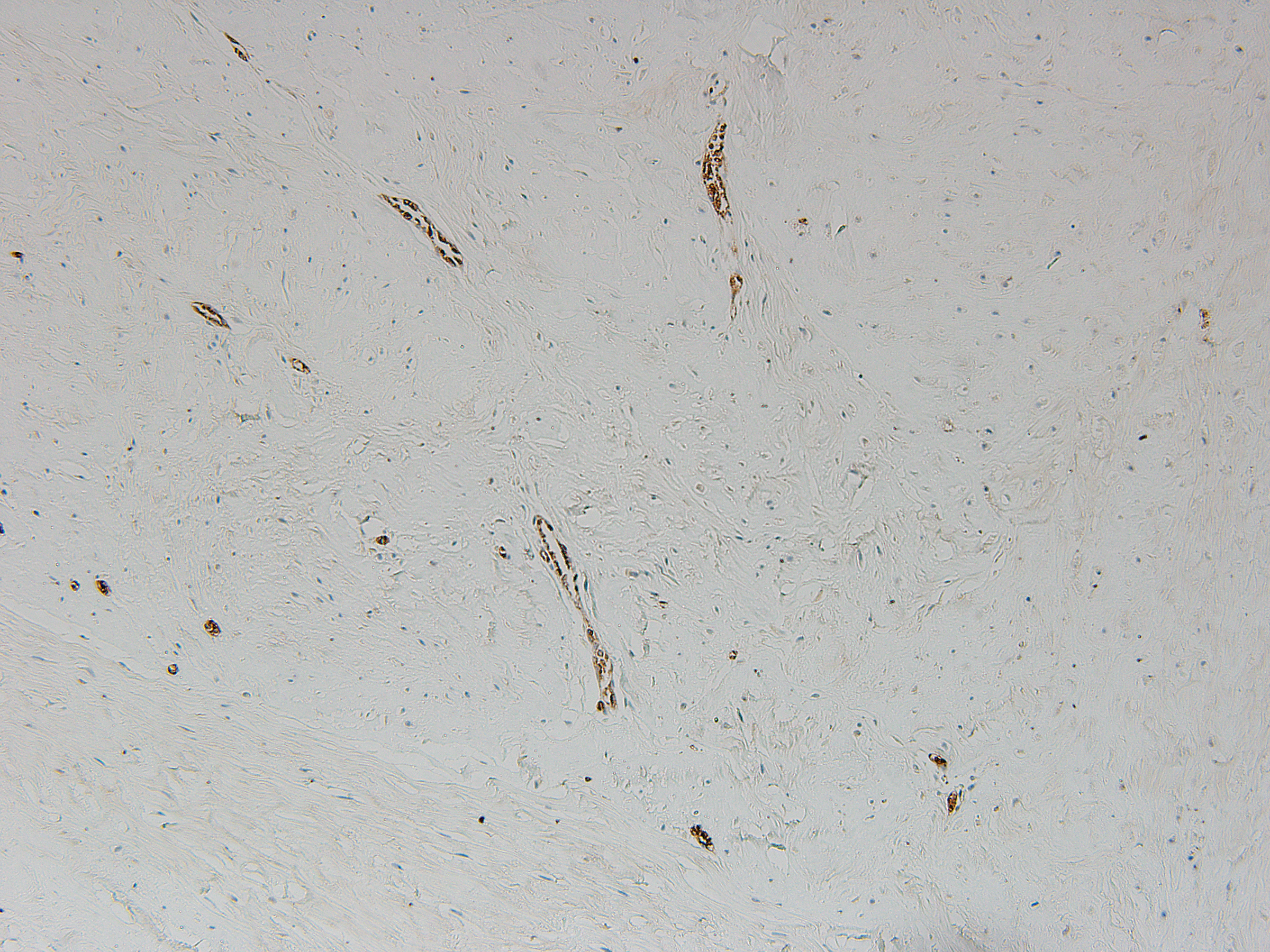

Supplement: Supplemental Material [file KBIE_A_2051838_SM7045.zip › supplementary document/images /Figure 4/CD34/EXO.tif]

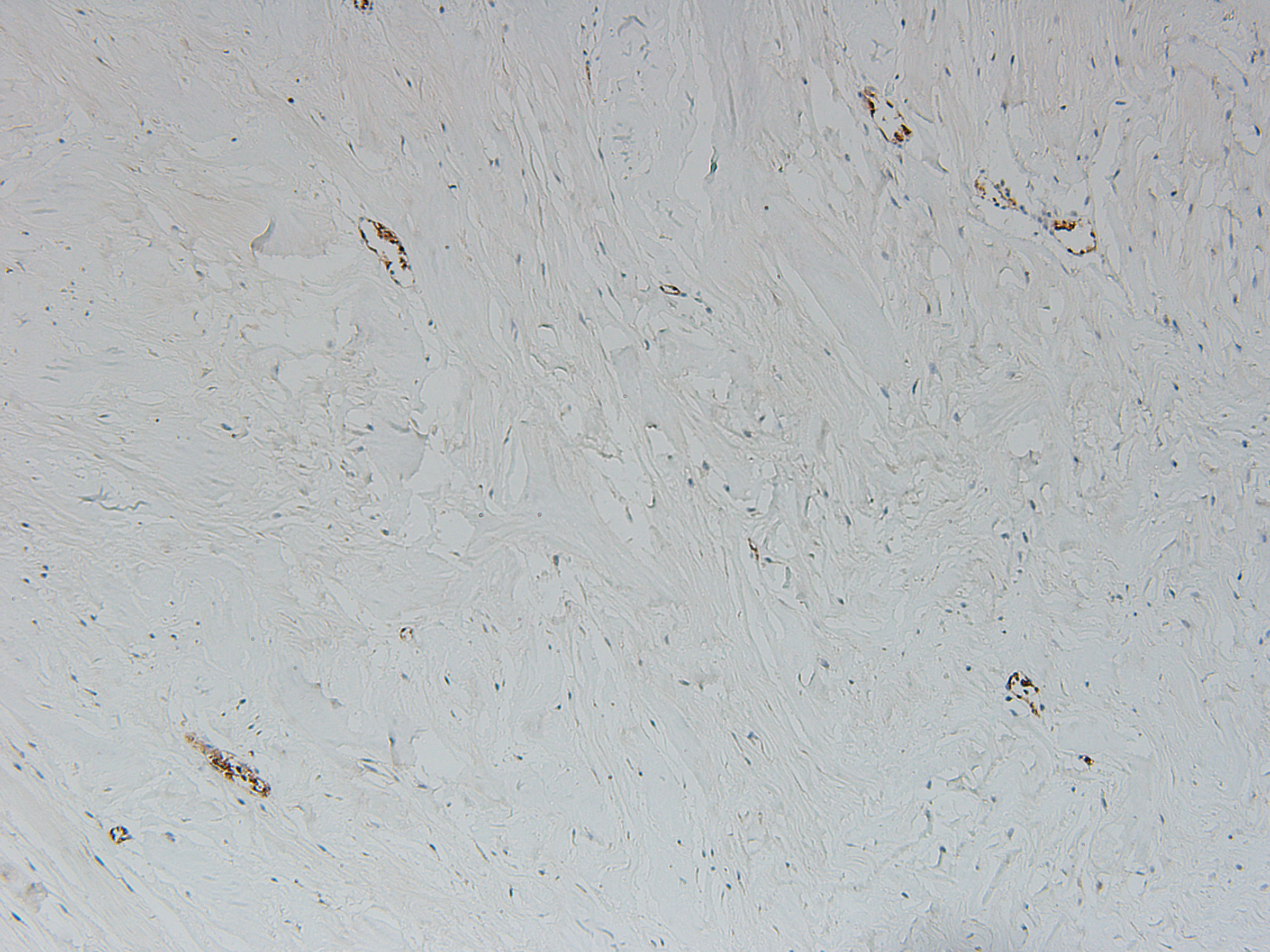

Supplement: Supplemental Material [file KBIE_A_2051838_SM7045.zip › supplementary document/images /Figure 4/CD34/AEFS.tif]

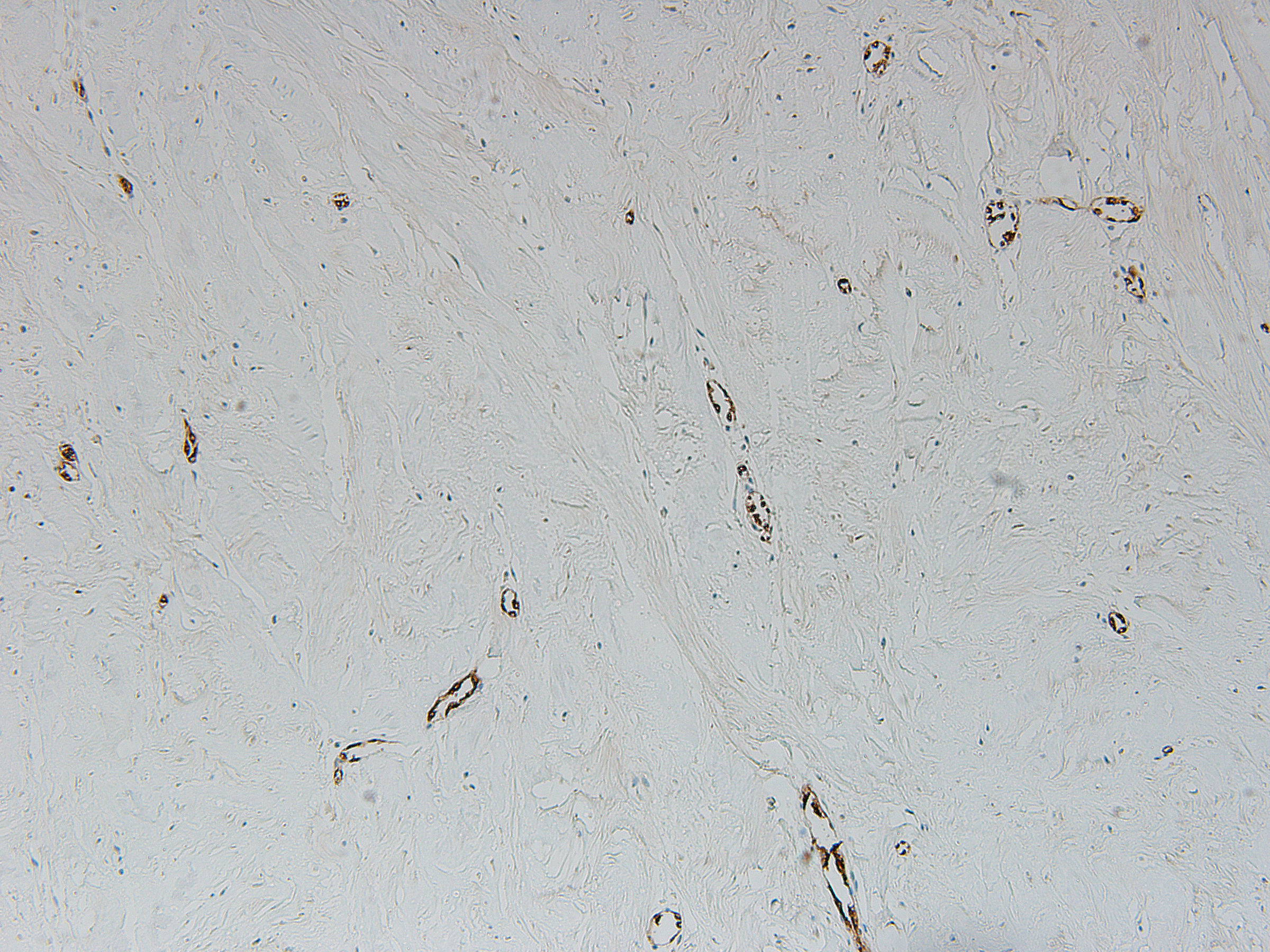

Supplement: Supplemental Material [file KBIE_A_2051838_SM7045.zip › supplementary document/images /Figure 4/CD34/PBS.tif]

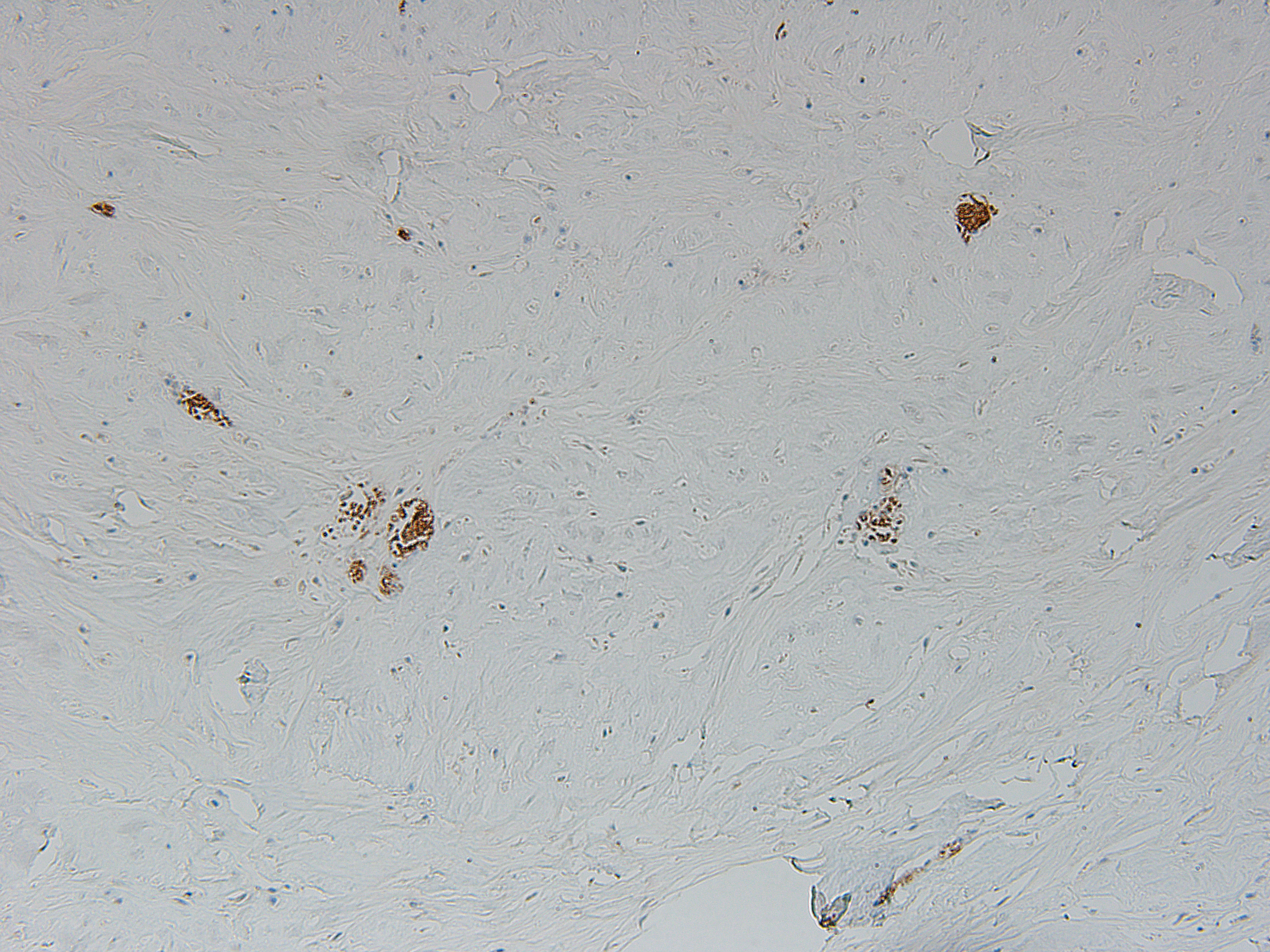

Supplement: Supplemental Material [file KBIE_A_2051838_SM7045.zip › supplementary document/images /Figure 4/CD31/EXO.tif]

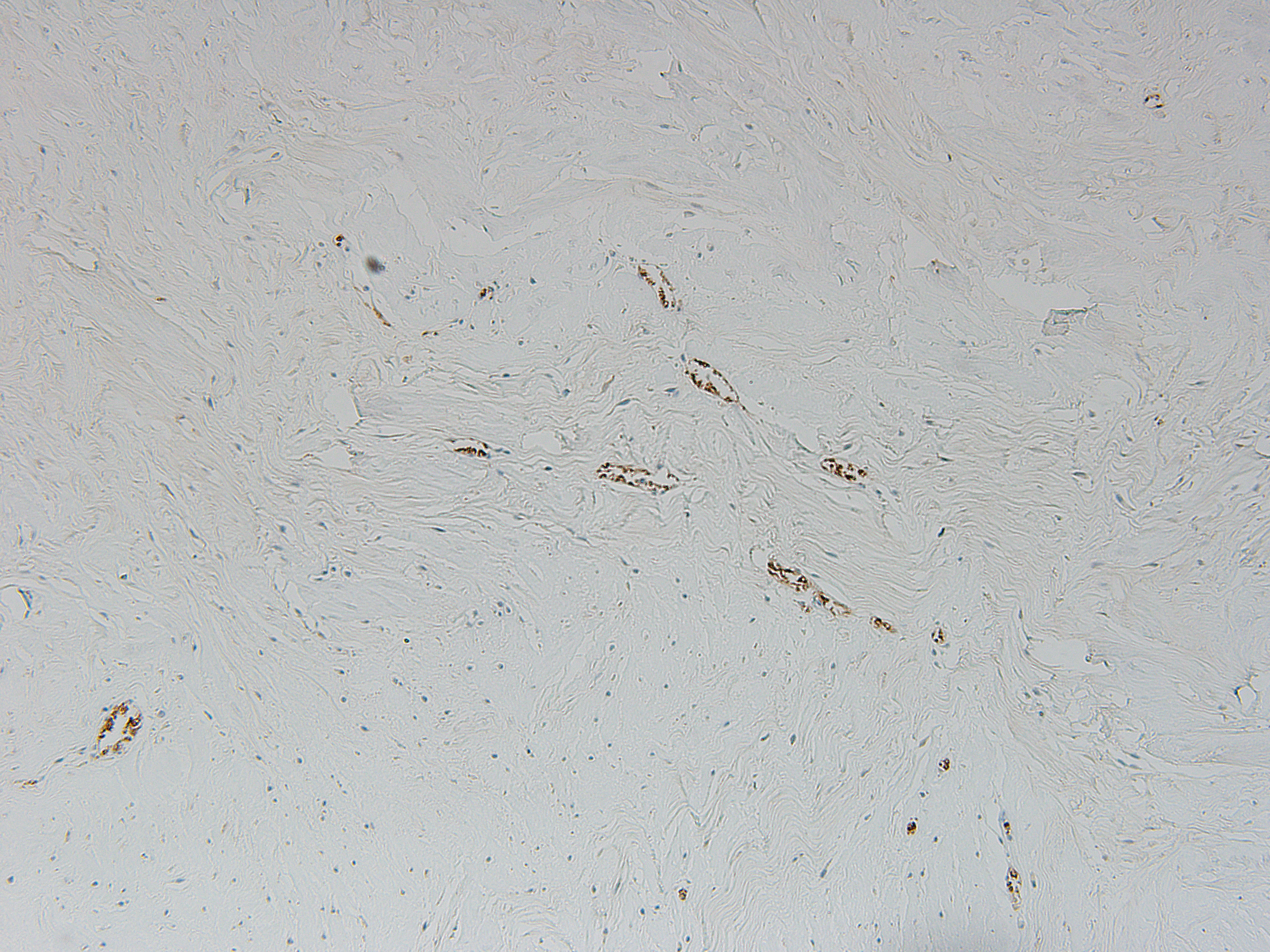

Supplement: Supplemental Material [file KBIE_A_2051838_SM7045.zip › supplementary document/images /Figure 4/CD31/AEFS.tif]

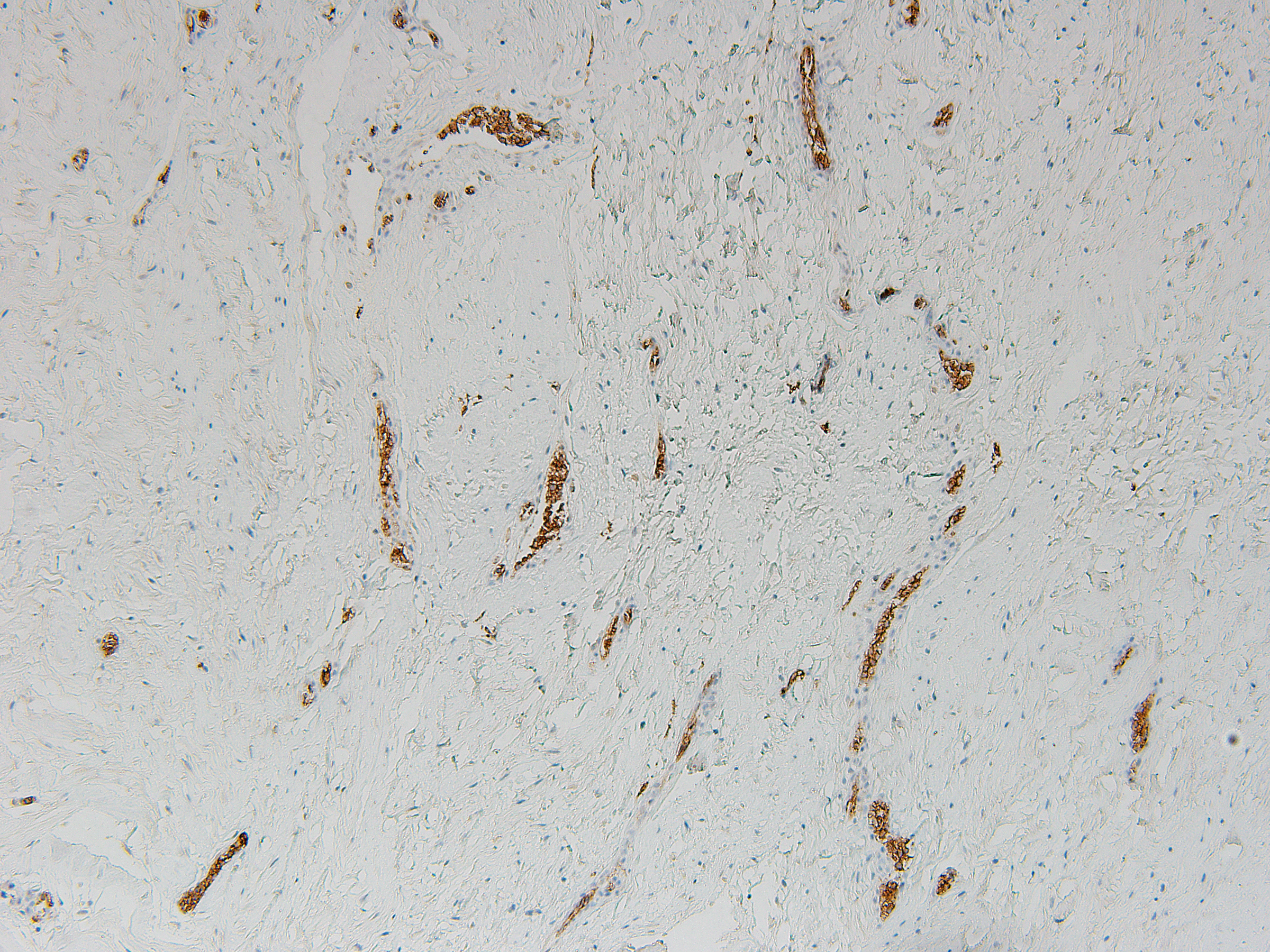

Supplement: Supplemental Material [file KBIE_A_2051838_SM7045.zip › supplementary document/images /Figure 4/CD31/PBS.tif]

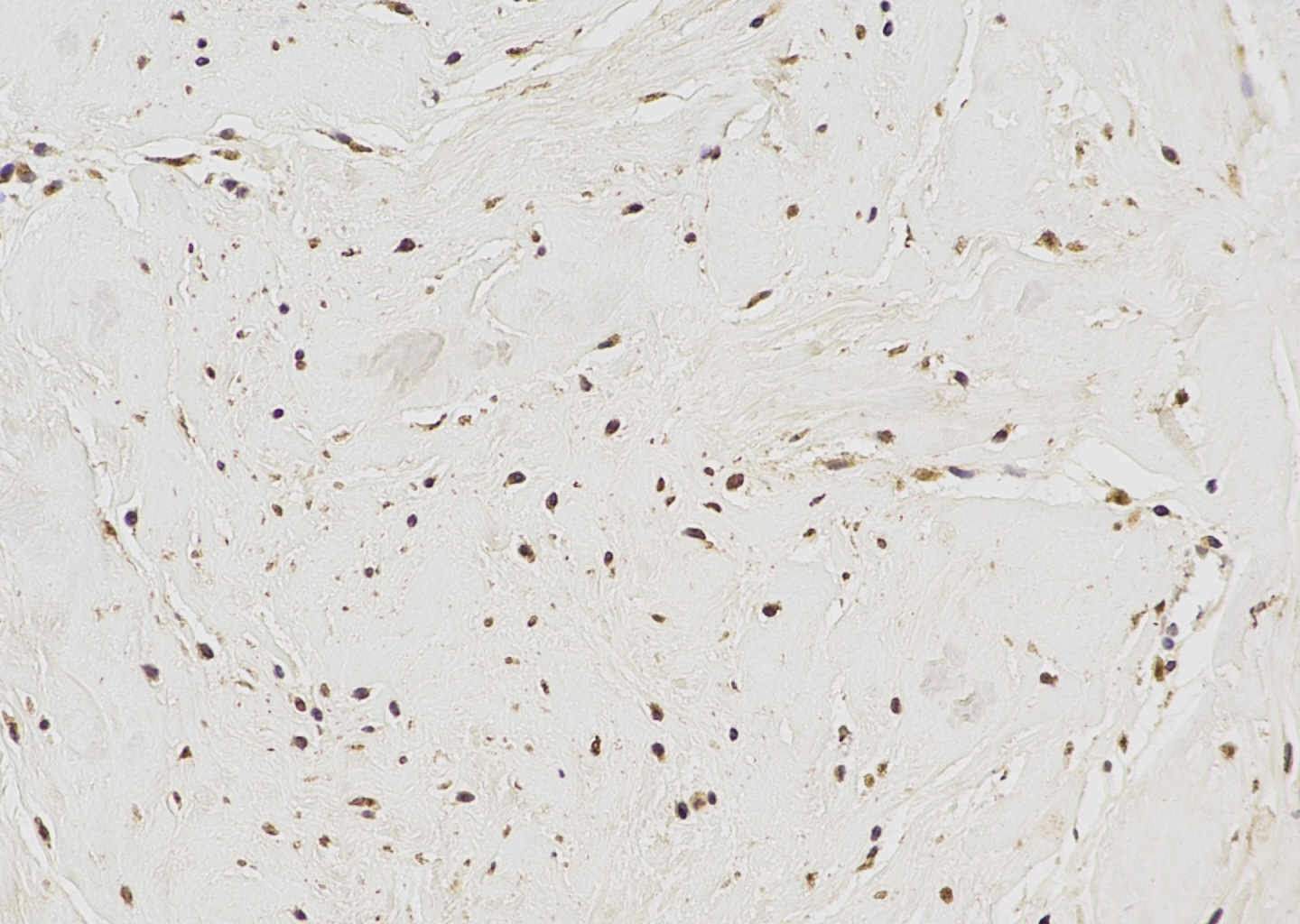

Supplement: Supplemental Material [file KBIE_A_2051838_SM7045.zip › supplementary document/images /Figure 4/COL-1/AEFS.jpg]

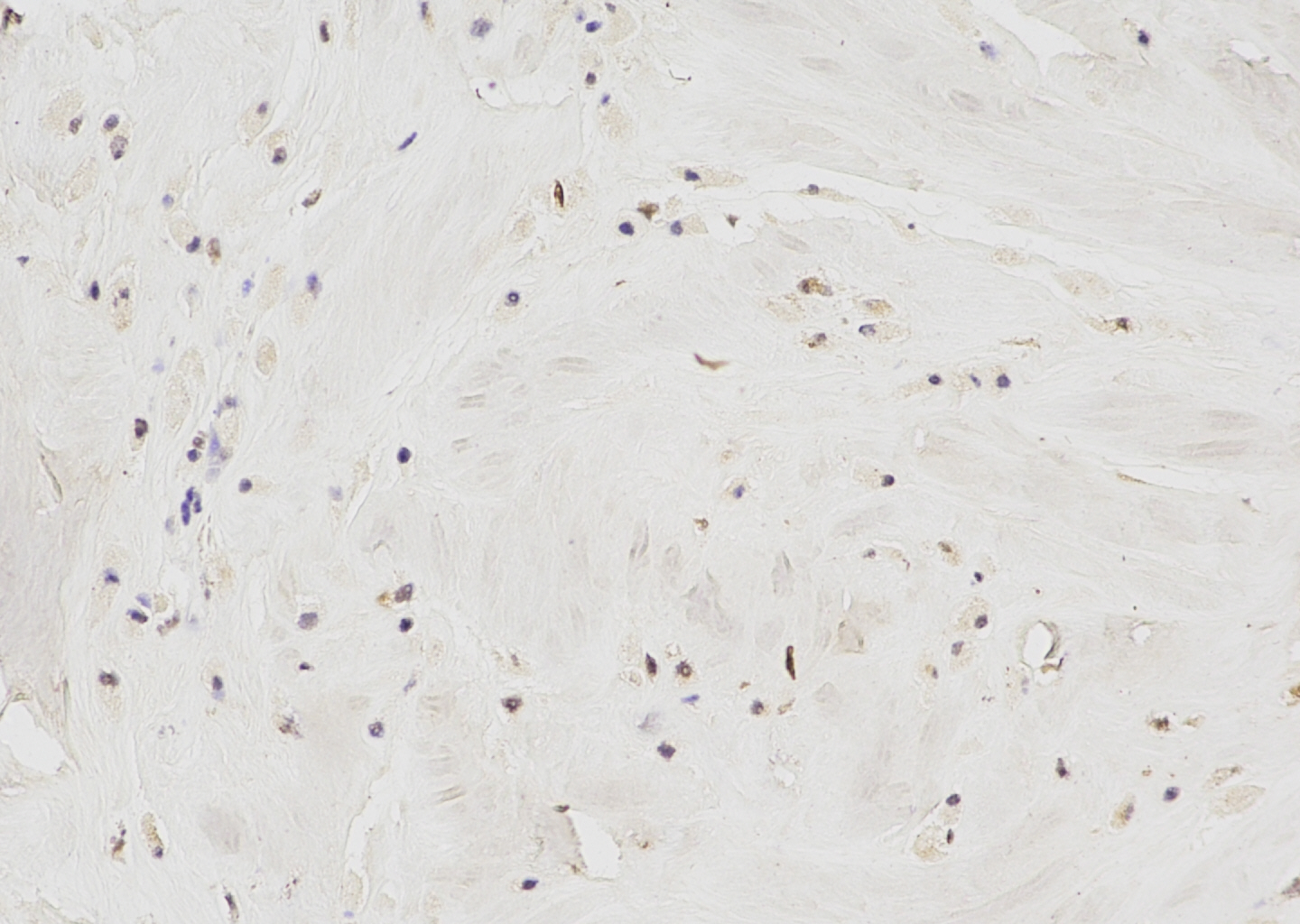

Supplement: Supplemental Material [file KBIE_A_2051838_SM7045.zip › supplementary document/images /Figure 4/COL-1/EXO.jpg]

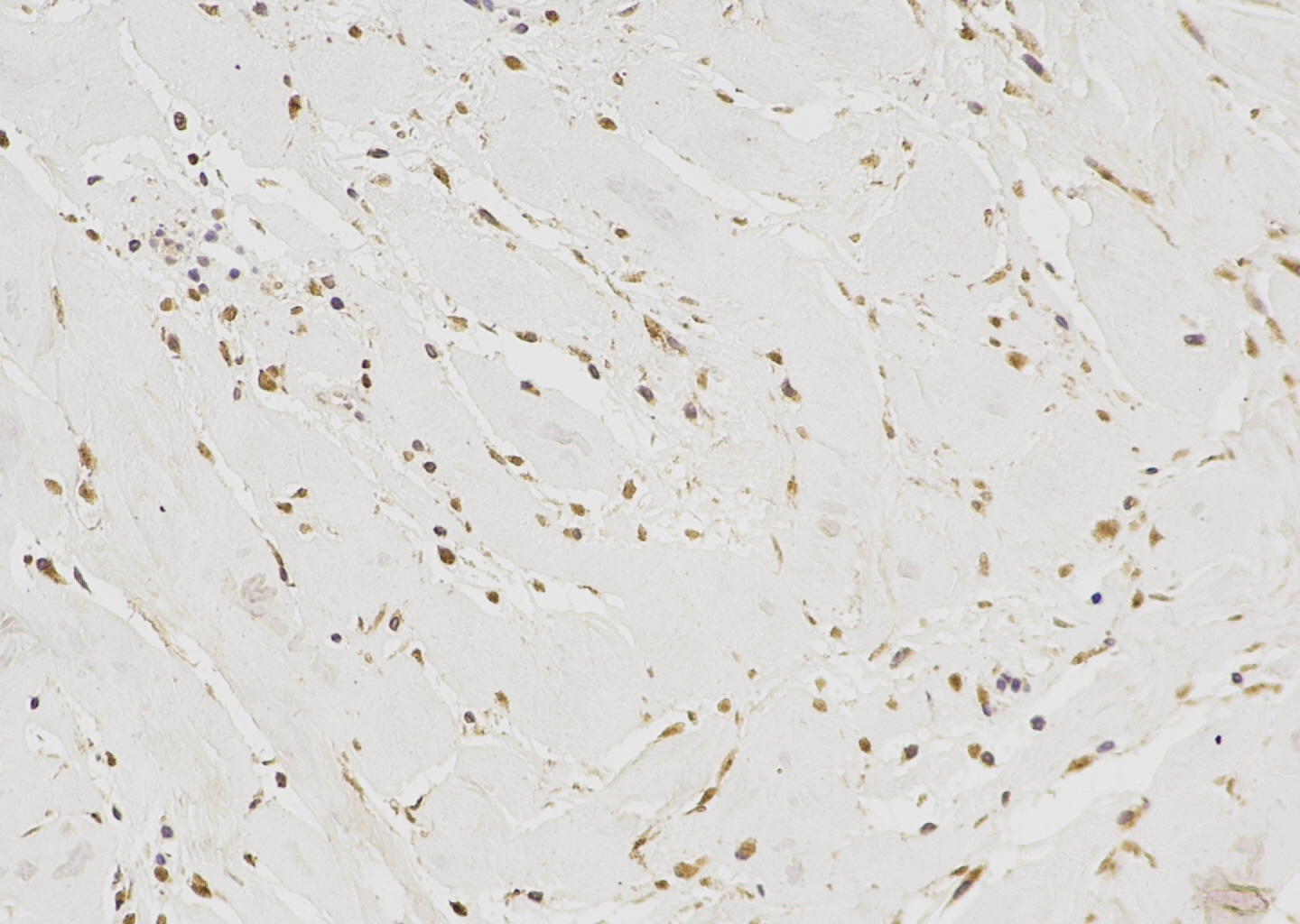

Supplement: Supplemental Material [file KBIE_A_2051838_SM7045.zip › supplementary document/images /Figure 4/COL-1/PBS.jpg]

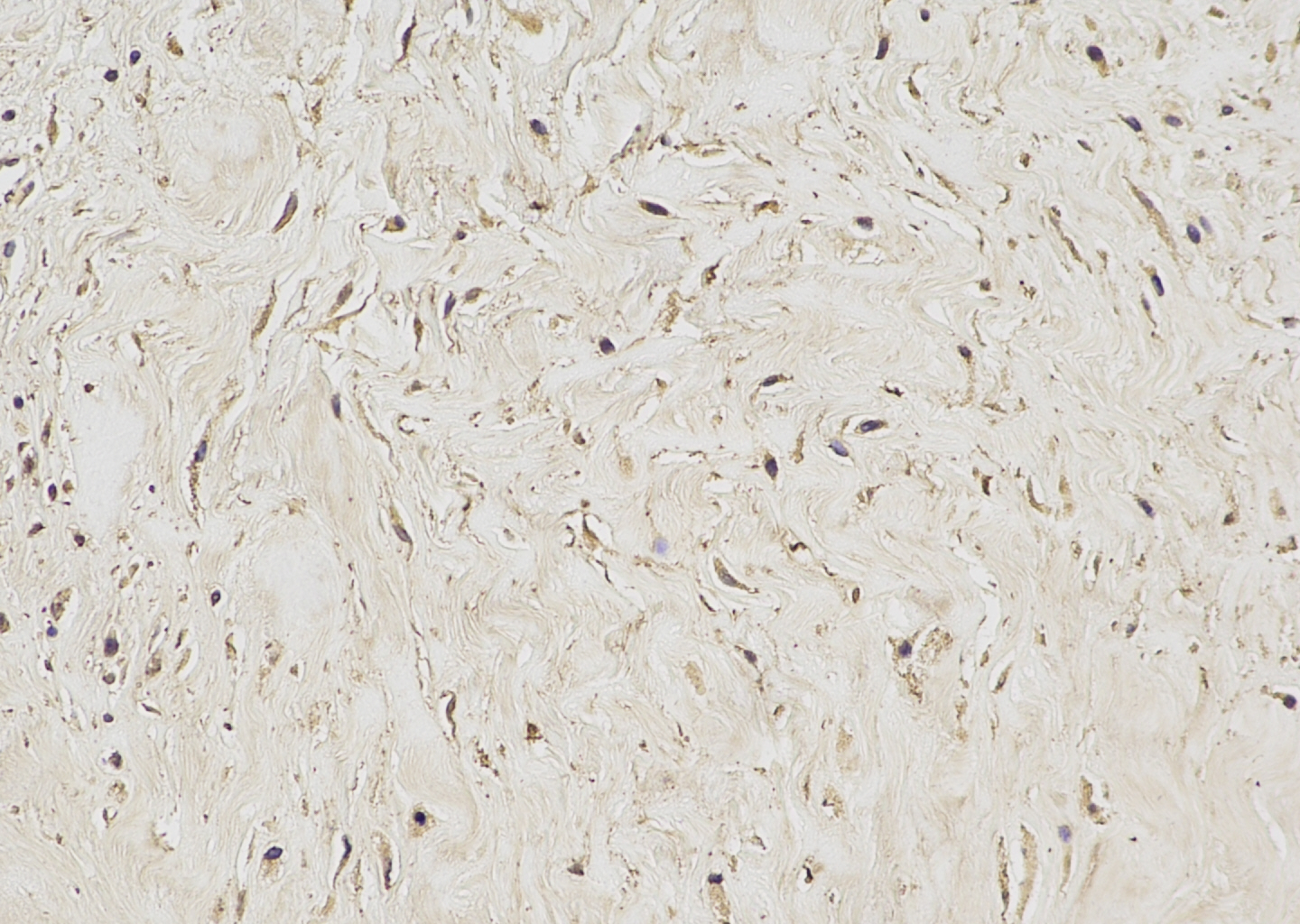

Supplement: Supplemental Material [file KBIE_A_2051838_SM7045.zip › supplementary document/images /Figure 4/FN/AEFS.jpg]

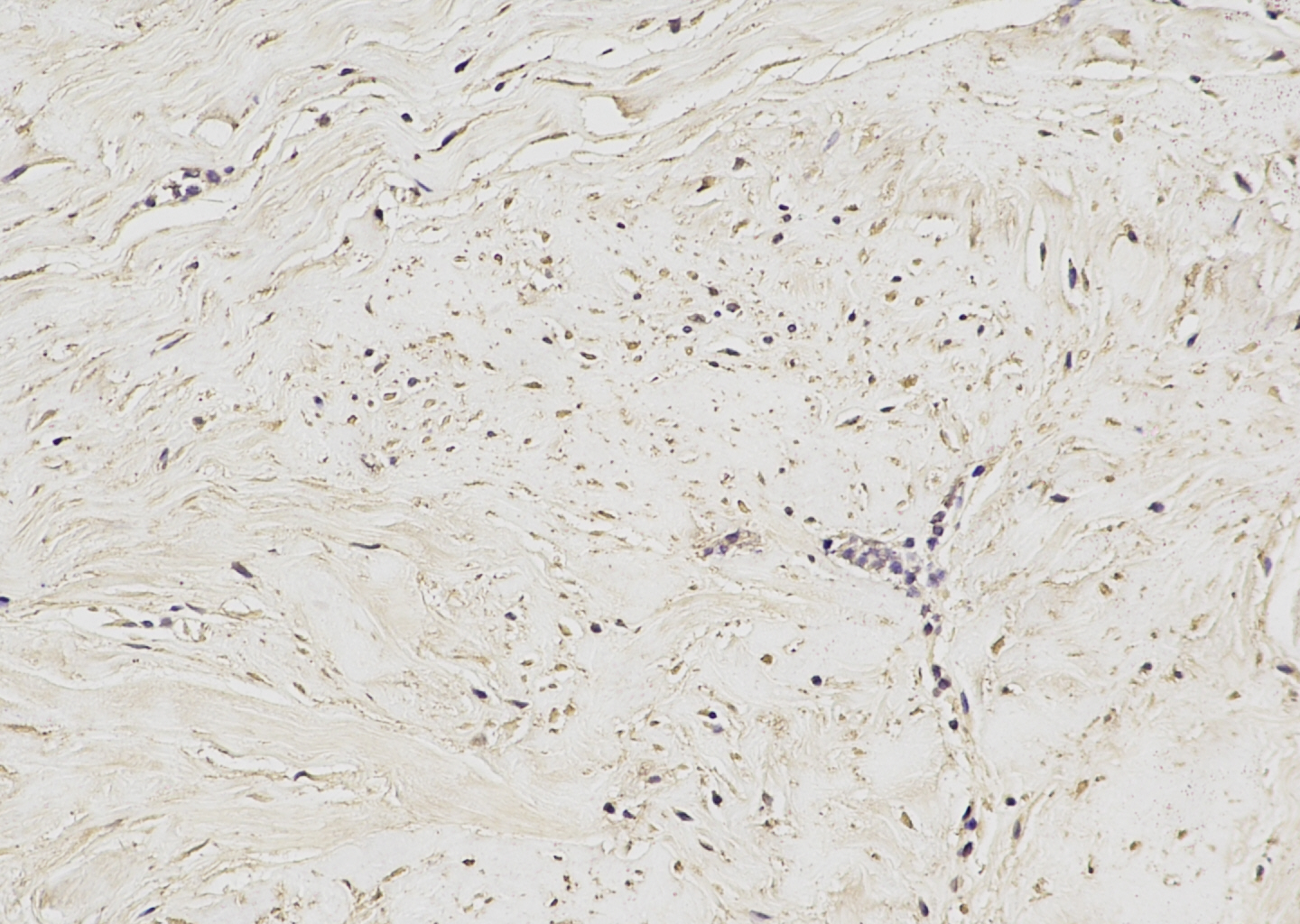

Supplement: Supplemental Material [file KBIE_A_2051838_SM7045.zip › supplementary document/images /Figure 4/FN/EXO.jpg]

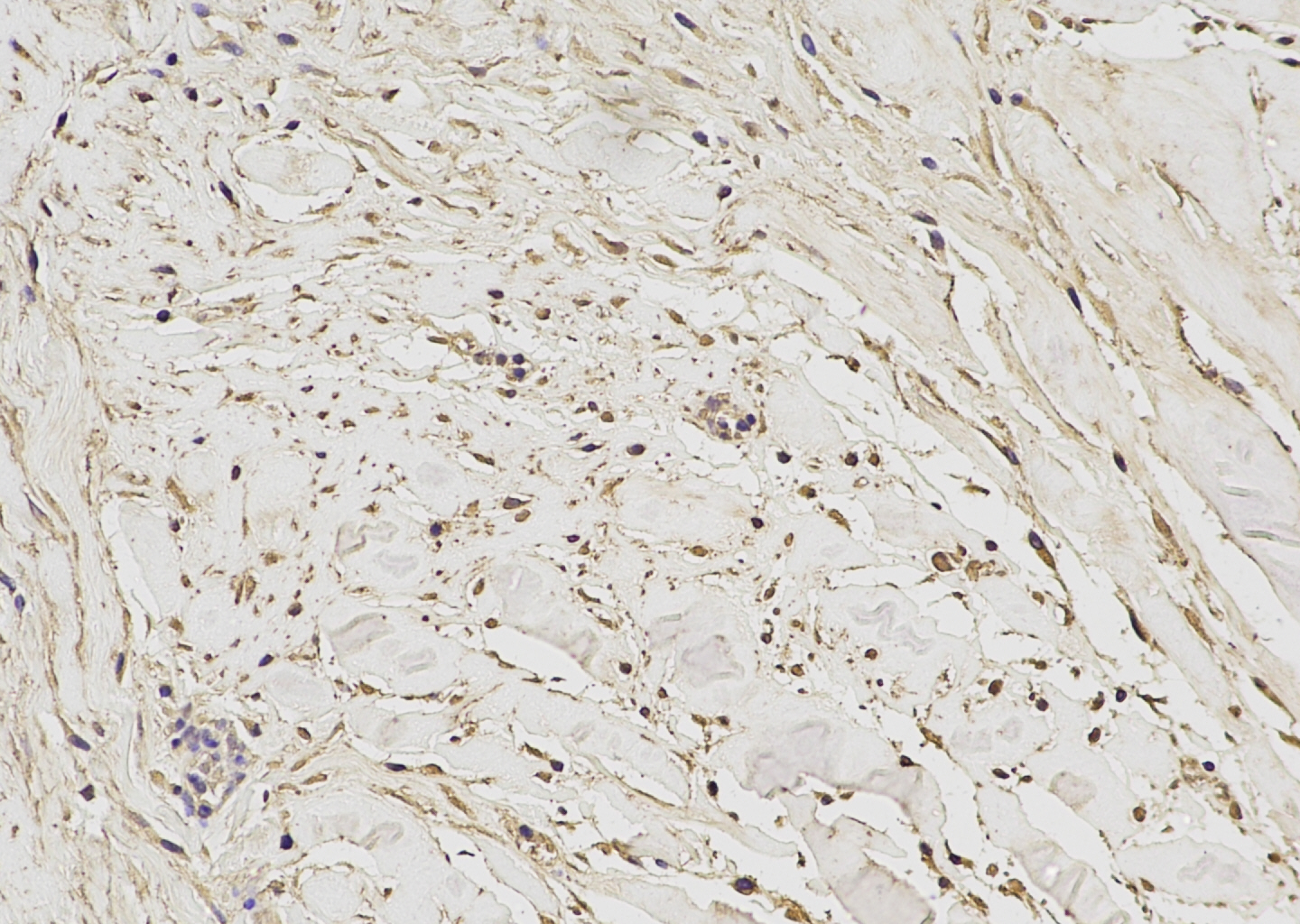

Supplement: Supplemental Material [file KBIE_A_2051838_SM7045.zip › supplementary document/images /Figure 4/FN/PBS.jpg]

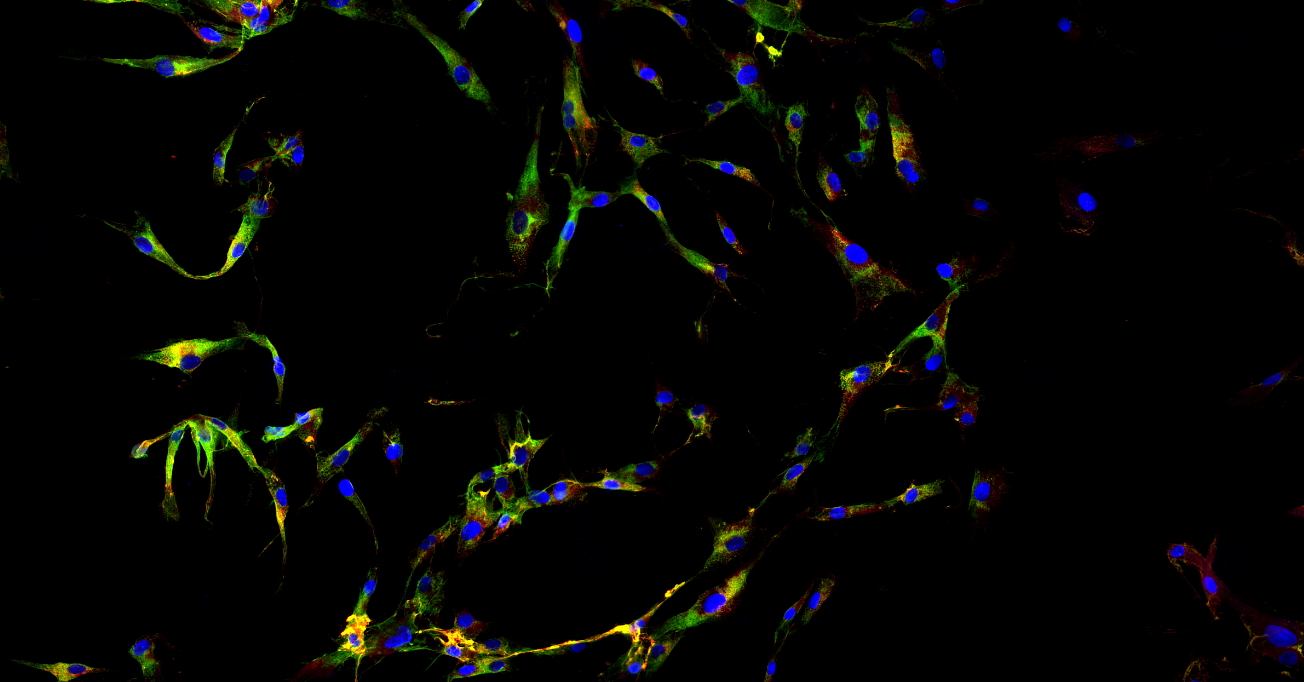

Supplement: Supplemental Material [file KBIE_A_2051838_SM7045.zip › supplementary document/images /Figure 3/EXO/Merge.jpg]

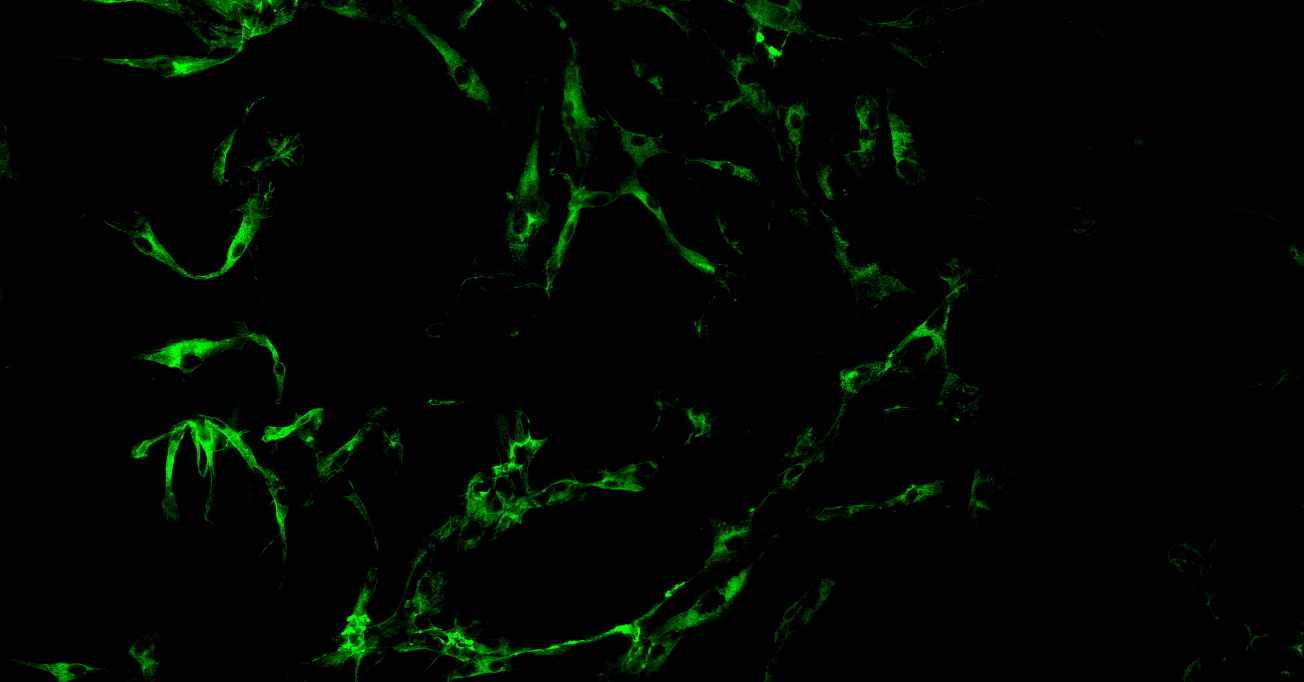

Supplement: Supplemental Material [file KBIE_A_2051838_SM7045.zip › supplementary document/images /Figure 3/EXO/FN.jpg]

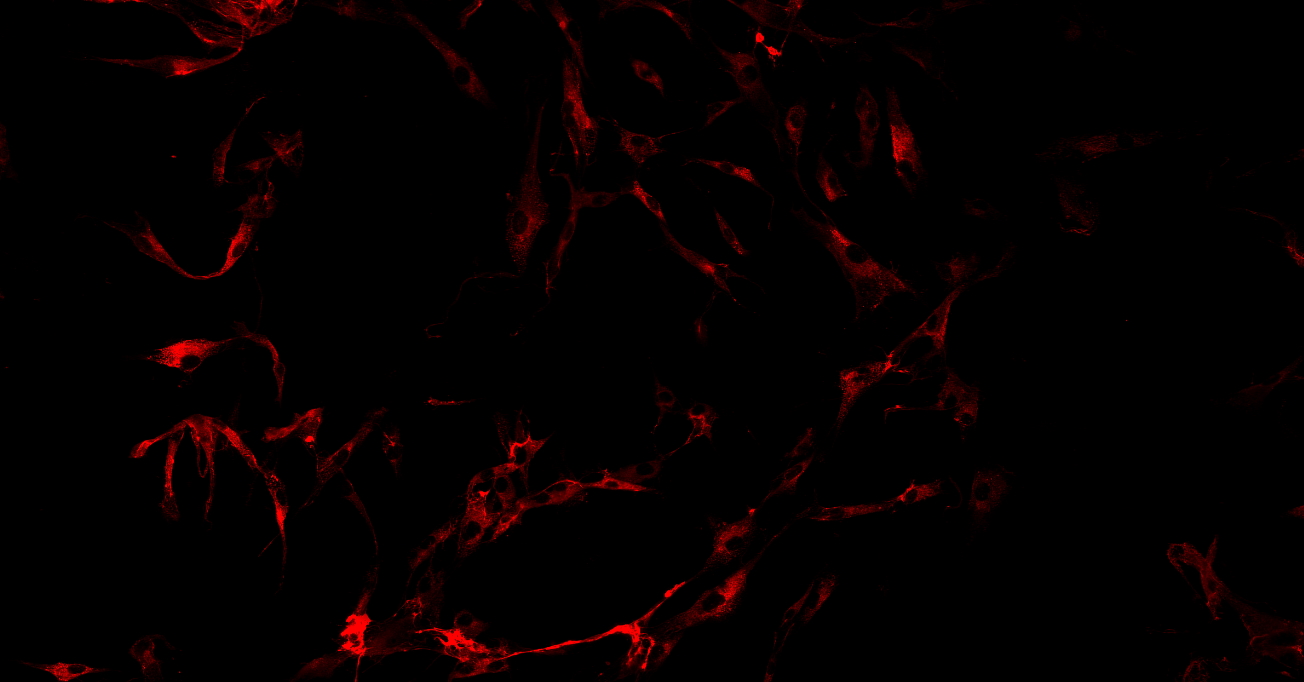

Supplement: Supplemental Material [file KBIE_A_2051838_SM7045.zip › supplementary document/images /Figure 3/EXO/COL-1.jpg]

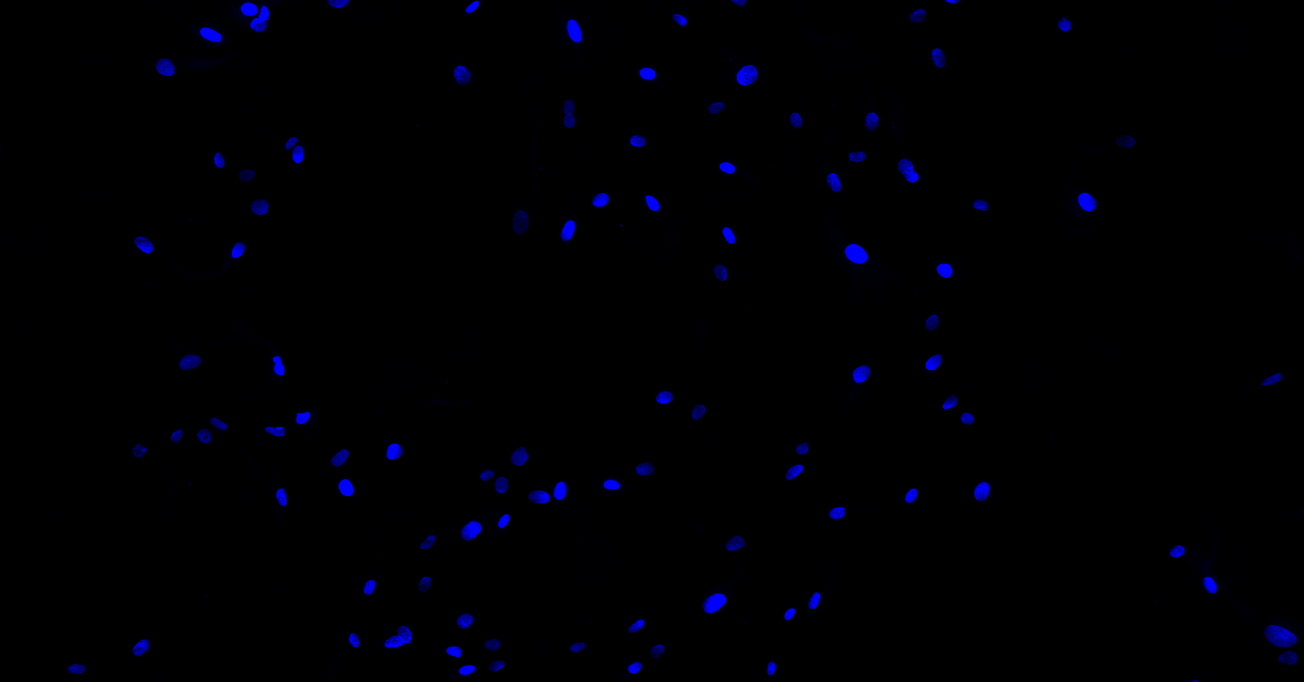

Supplement: Supplemental Material [file KBIE_A_2051838_SM7045.zip › supplementary document/images /Figure 3/EXO/DAPI.jpg]

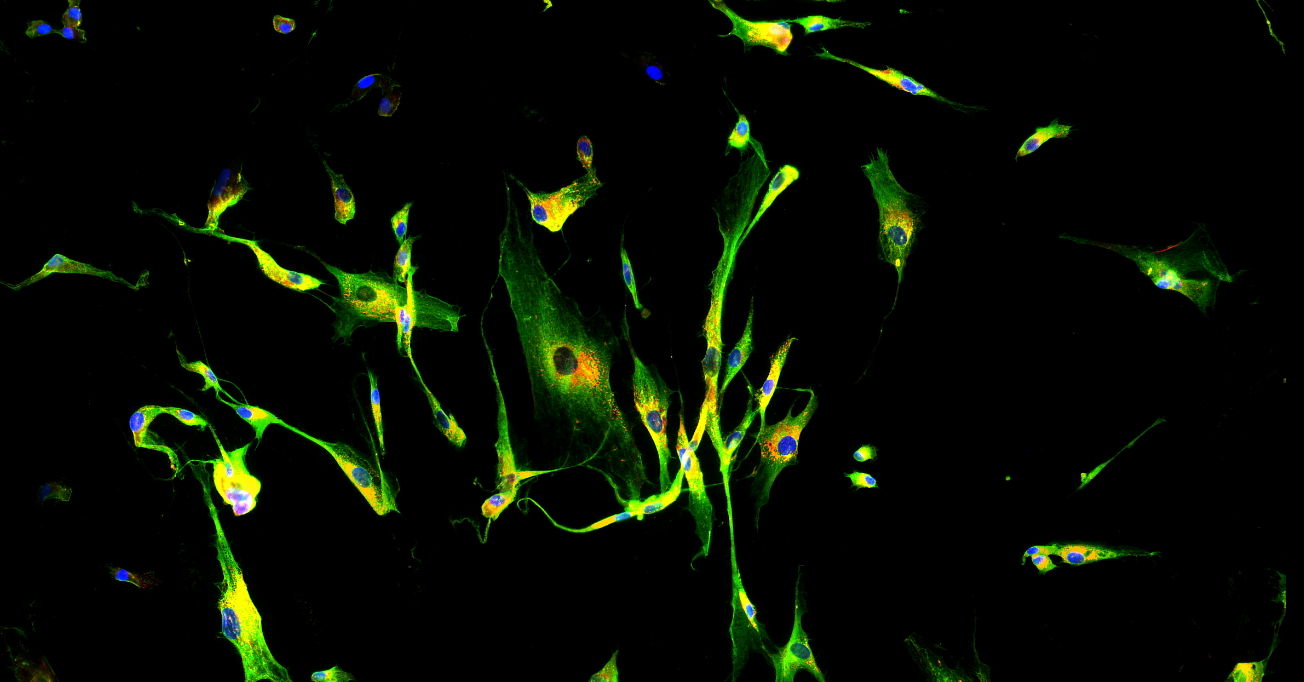

Supplement: Supplemental Material [file KBIE_A_2051838_SM7045.zip › supplementary document/images /Figure 3/PBS/Merge.jpg]

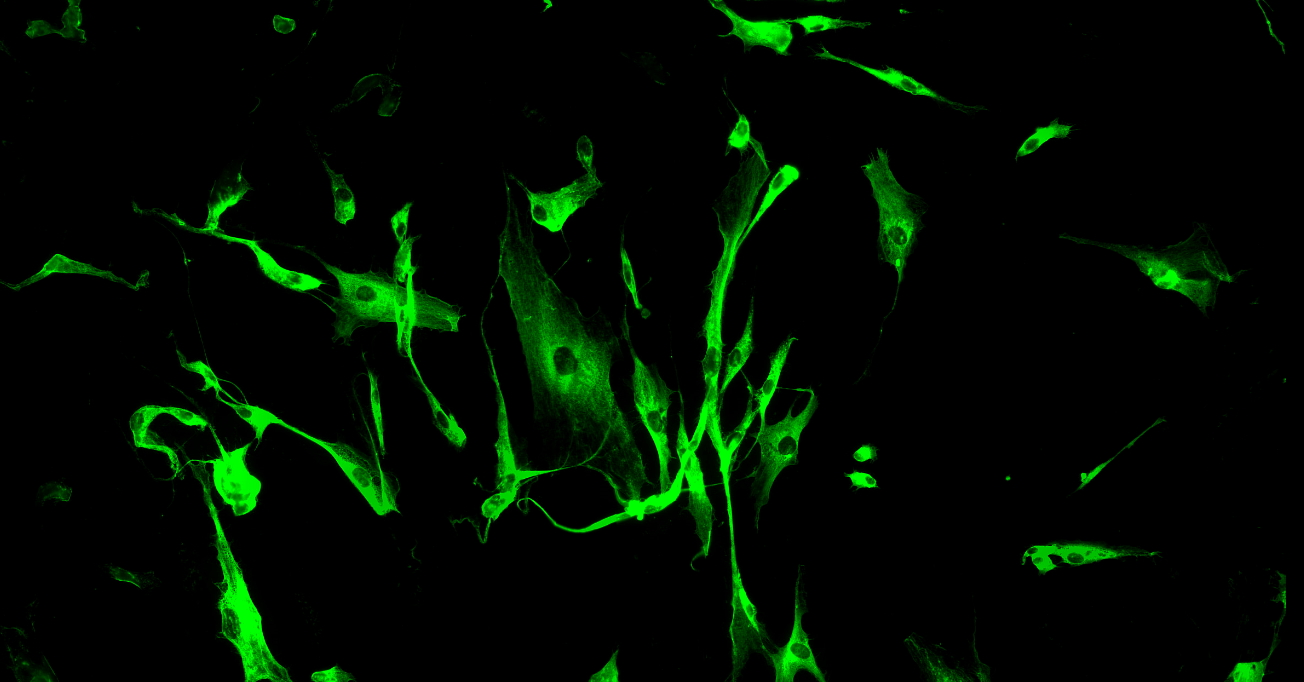

Supplement: Supplemental Material [file KBIE_A_2051838_SM7045.zip › supplementary document/images /Figure 3/PBS/FN.jpg]

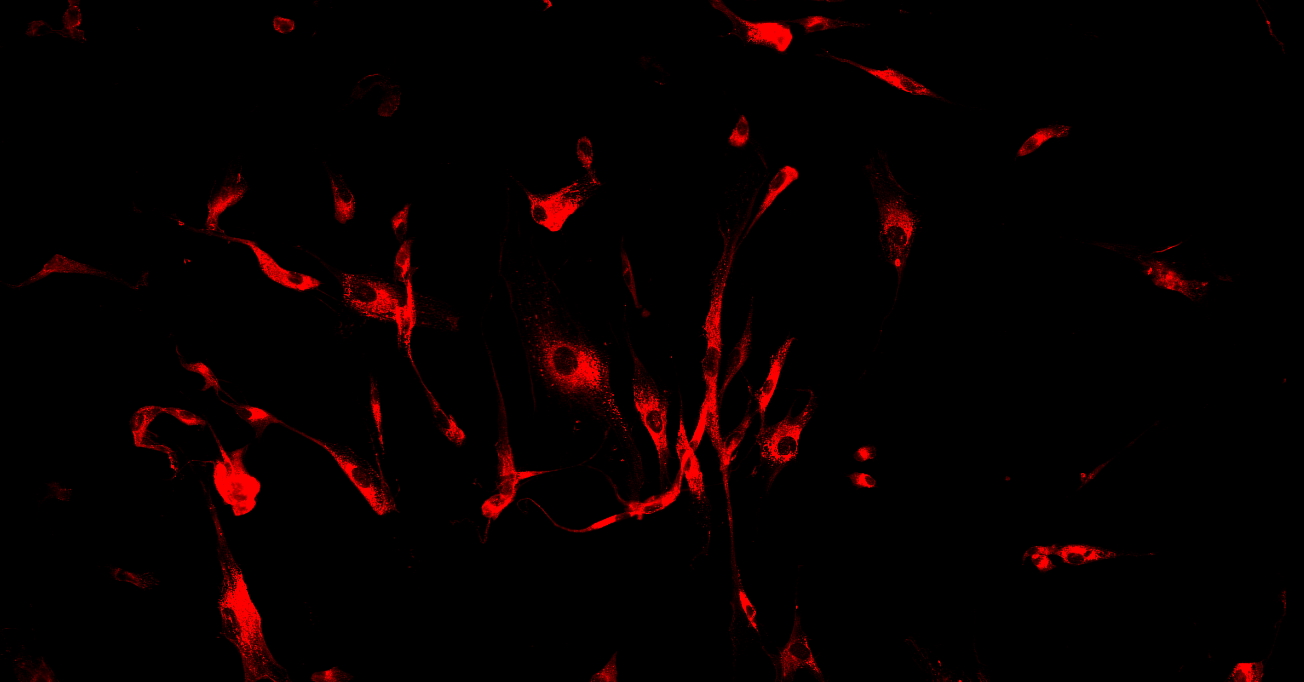

Supplement: Supplemental Material [file KBIE_A_2051838_SM7045.zip › supplementary document/images /Figure 3/PBS/COL-1.jpg]

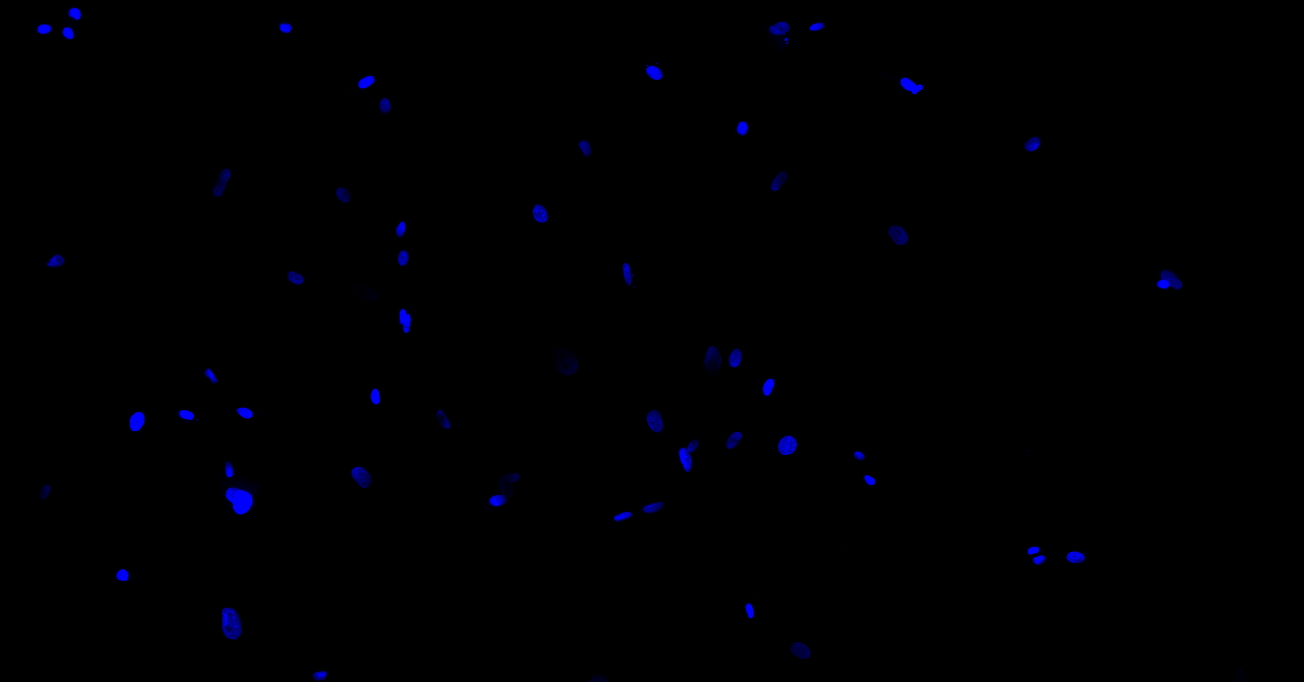

Supplement: Supplemental Material [file KBIE_A_2051838_SM7045.zip › supplementary document/images /Figure 3/PBS/DAPI.jpg]

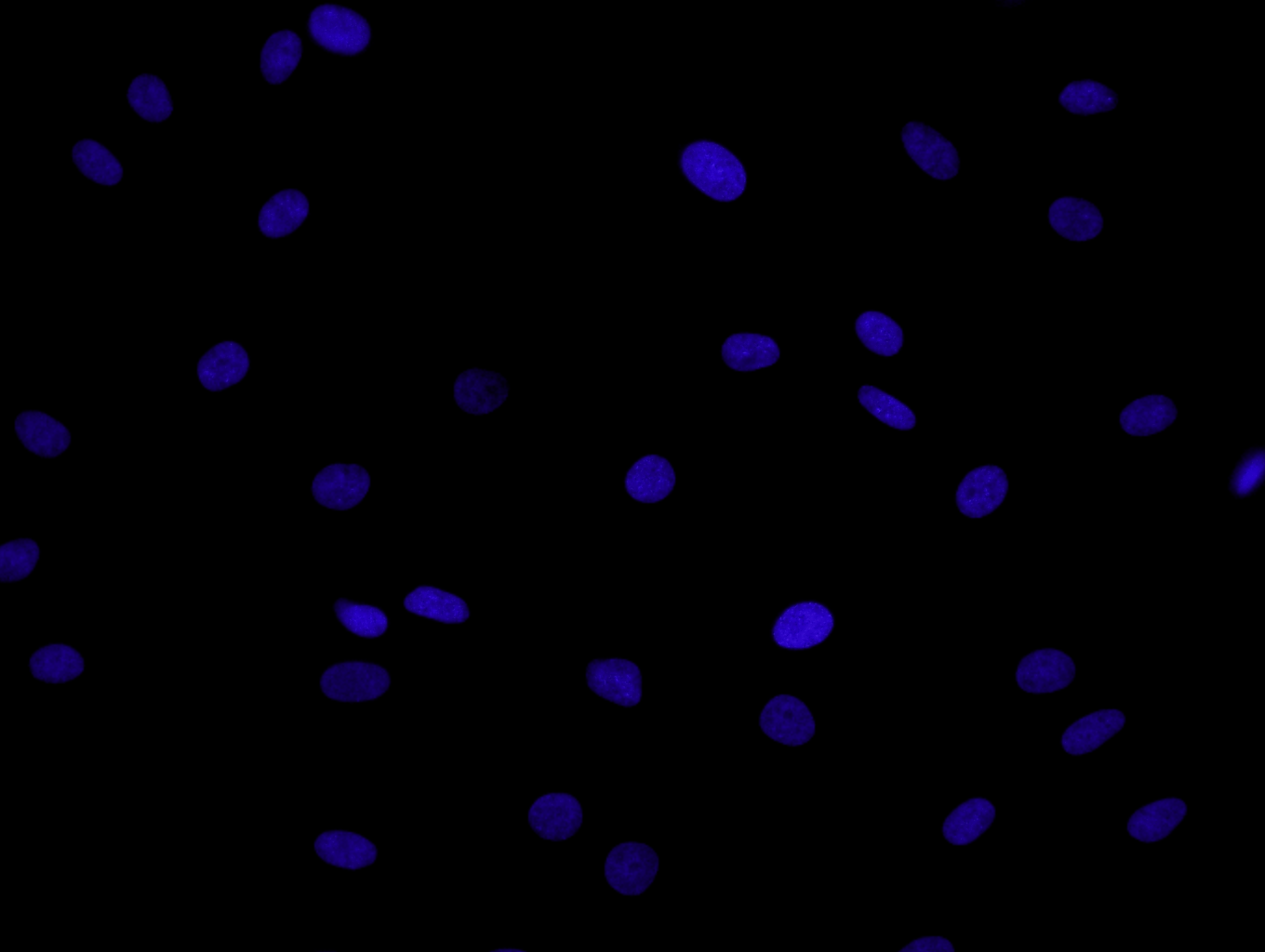

Supplement: Supplemental Material [file KBIE_A_2051838_SM7045.zip › supplementary document/images /Figure 2/C/0H/DAPI.tif]

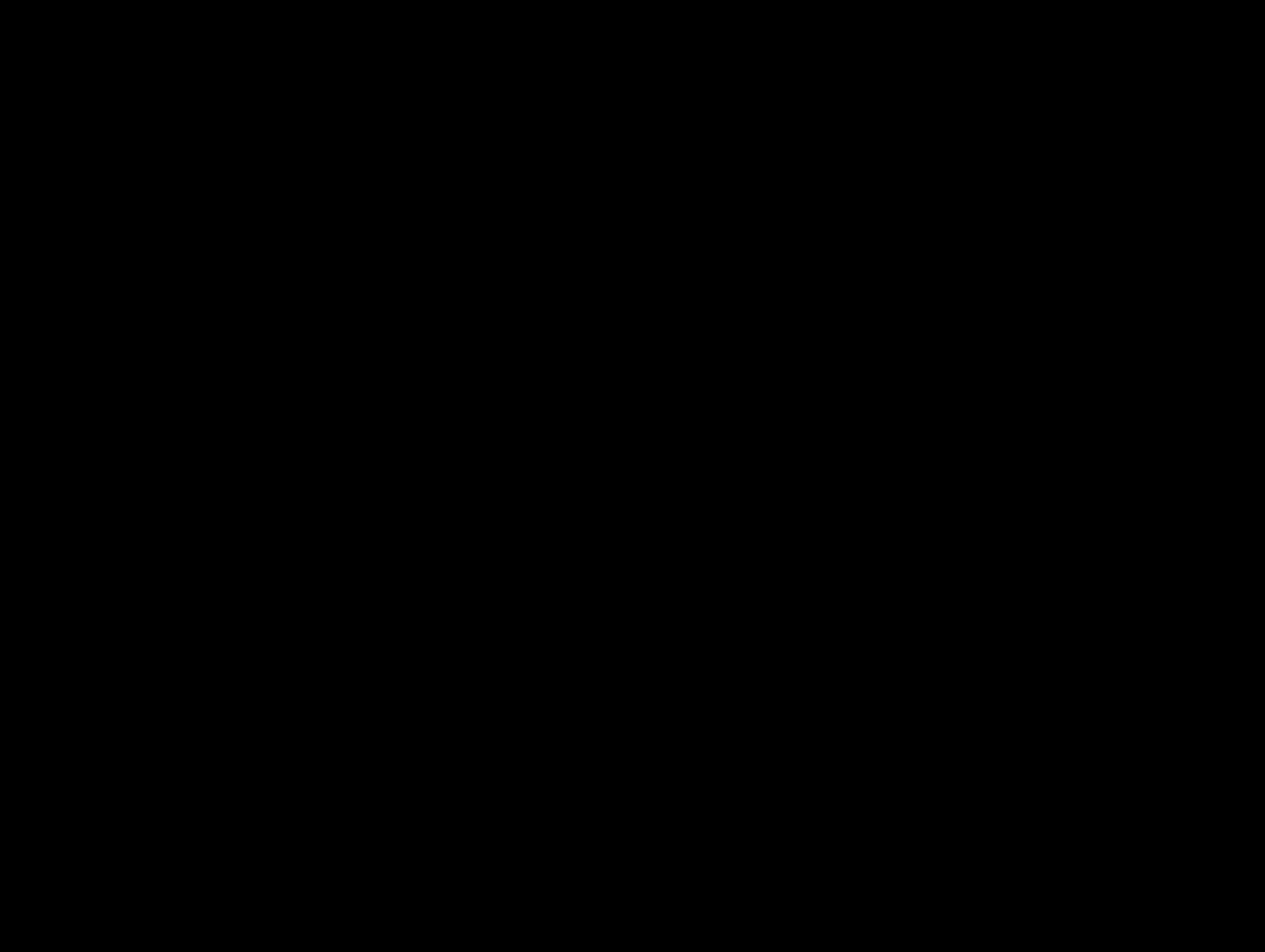

Supplement: Supplemental Material [file KBIE_A_2051838_SM7045.zip › supplementary document/images /Figure 2/C/0H/PKH67.tif]

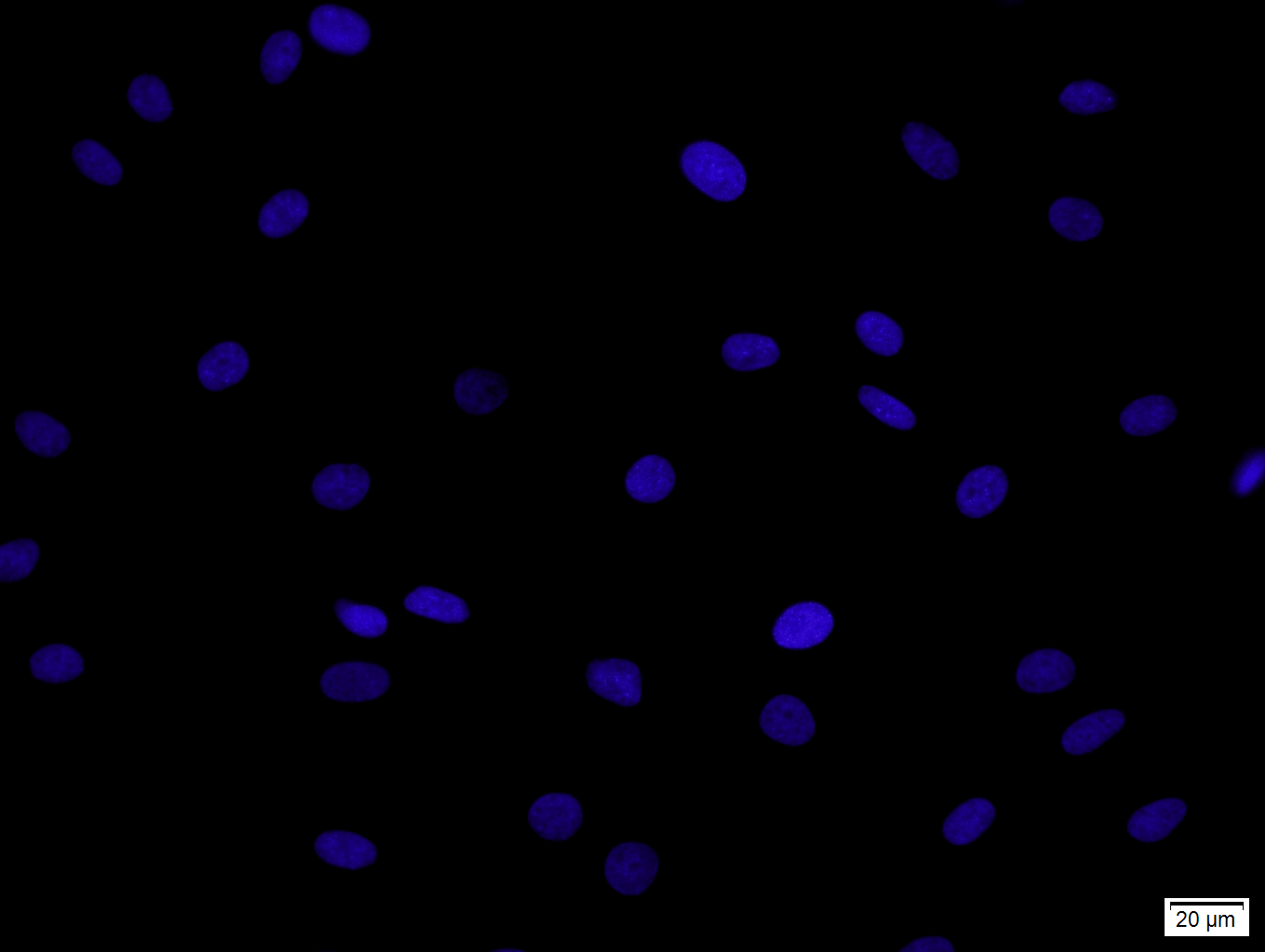

Supplement: Supplemental Material [file KBIE_A_2051838_SM7045.zip › supplementary document/images /Figure 2/C/0H/Merge.tif]

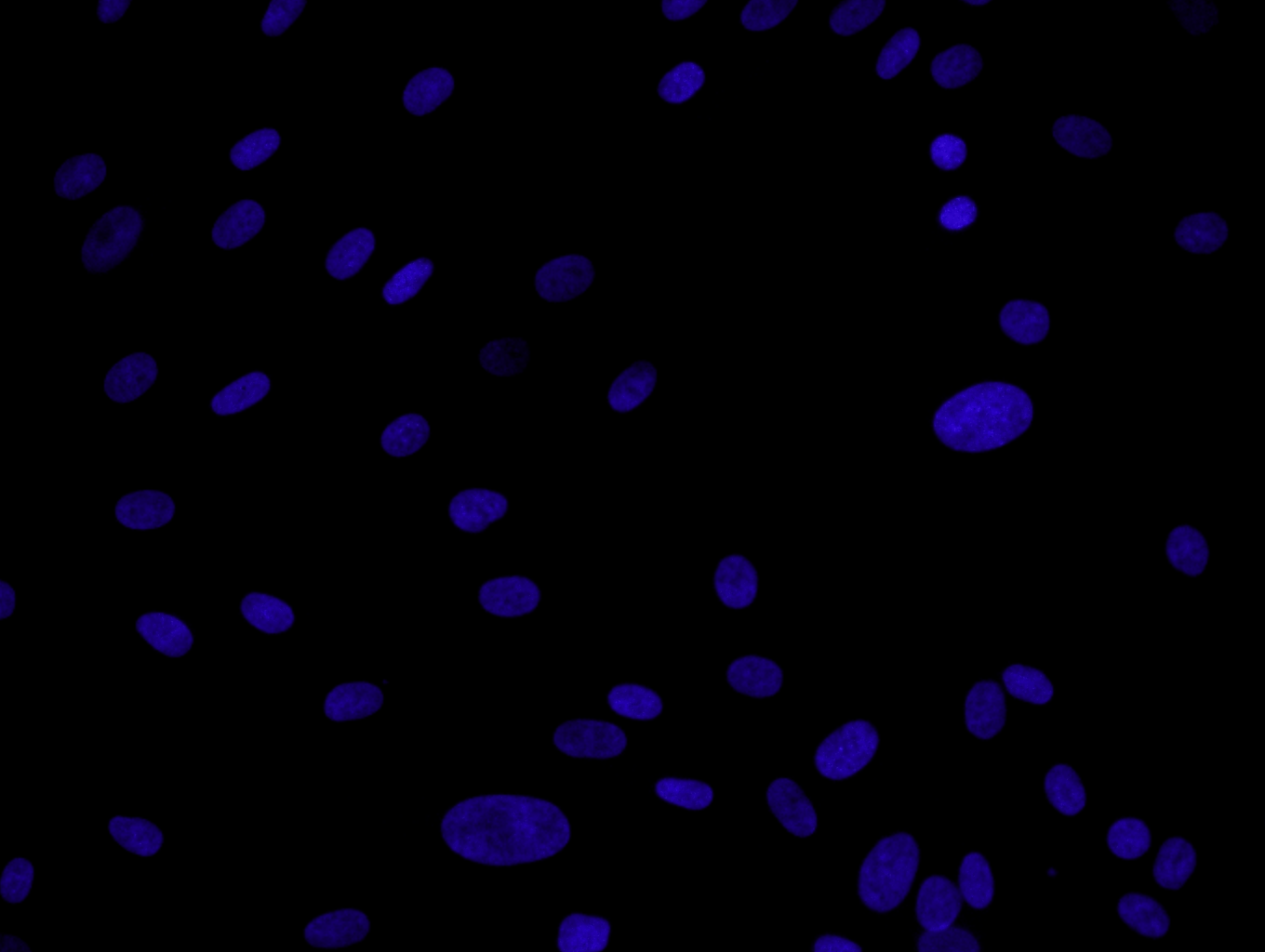

Supplement: Supplemental Material [file KBIE_A_2051838_SM7045.zip › supplementary document/images /Figure 2/C/24H/DAPI.tif]

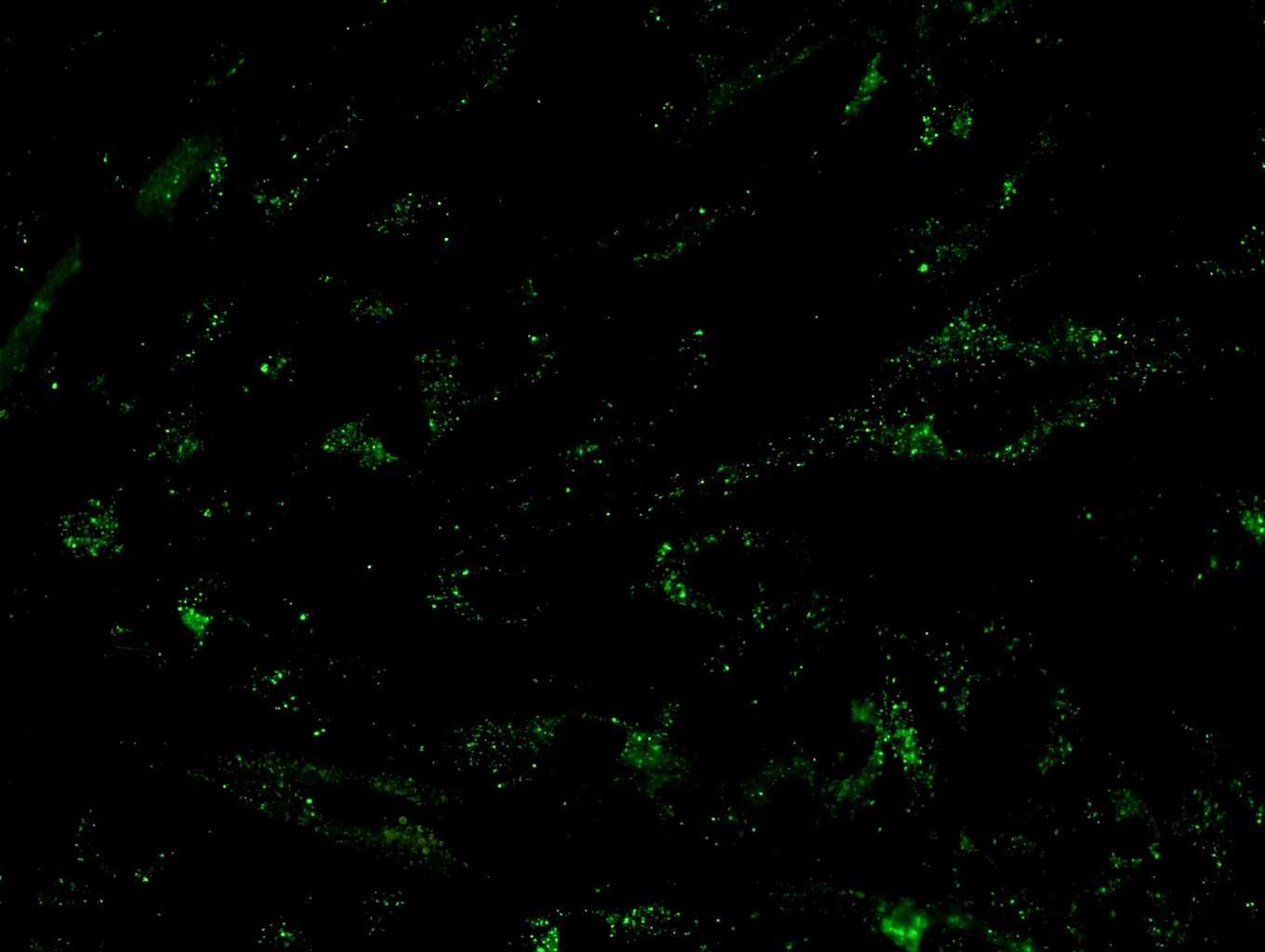

Supplement: Supplemental Material [file KBIE_A_2051838_SM7045.zip › supplementary document/images /Figure 2/C/24H/PKH67.tif]

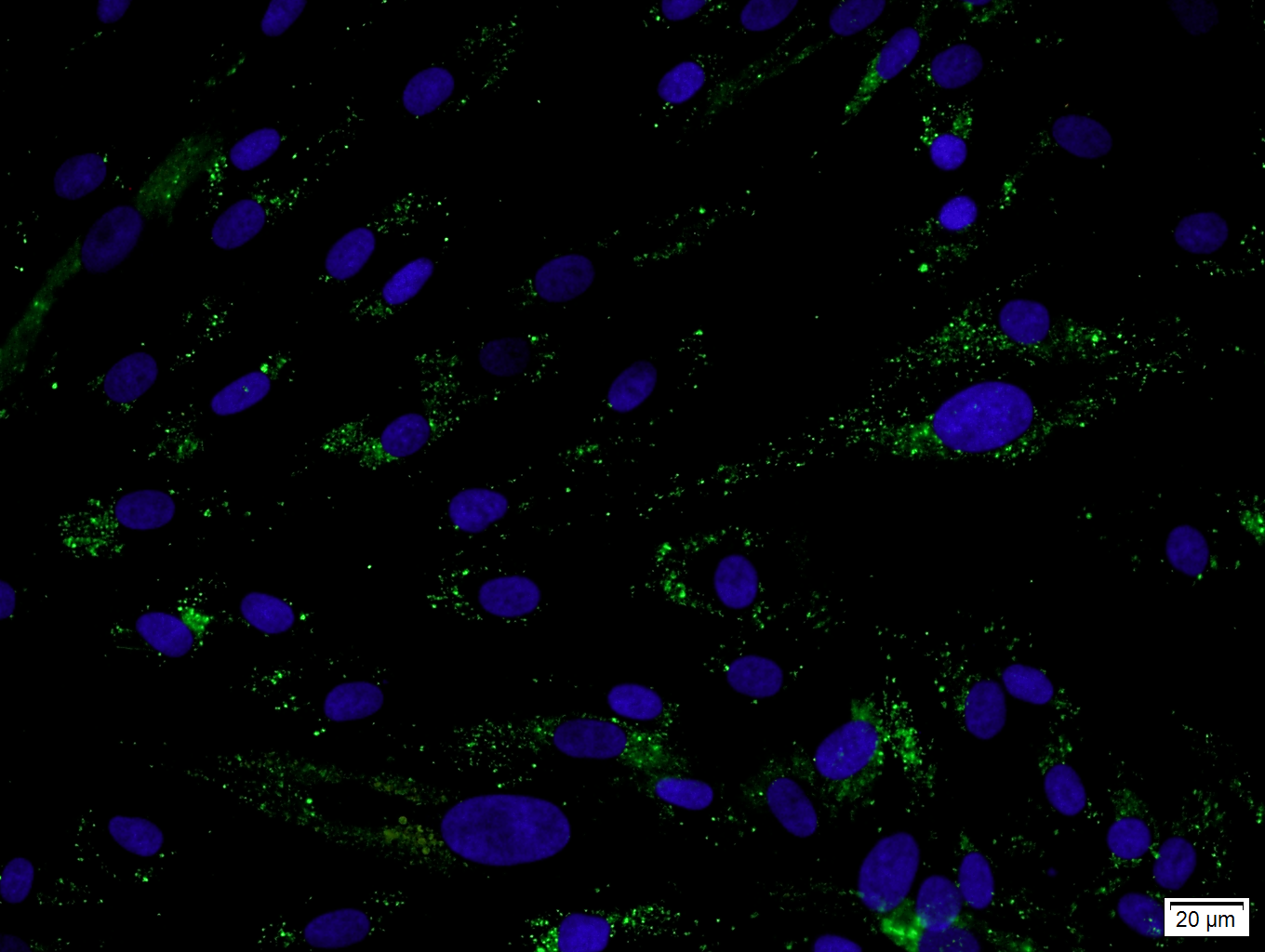

Supplement: Supplemental Material [file KBIE_A_2051838_SM7045.zip › supplementary document/images /Figure 2/C/24H/Merge.tif]
